# Time-Based and Event-Based Prospective Memory in Mild Cognitive Impairment and Alzheimer’s Disease Patients: A Systematic Review and Meta-analysis

**DOI:** 10.1007/s11065-023-09626-y

**Published:** 2023-11-14

**Authors:** Rafael Román-Caballero, Giovanna Mioni

**Affiliations:** 1 Mind, Brain and Behavior Research Center (CIMCYC), University of Granada, Granada, Spain; 2 Department of Experimental Psychology, University of Granada, Granada, Spain; 3 Department of General Psychology, University of Padova, Via Venezia 8, 35121 Padua, Italy

**Keywords:** Prospective memory, Event-based prospective memory, Time-based prospective memory, Neurodegenerative condition, Impaired cognitive functions, Pathological ageing

## Abstract

Prospective memory (PM) is the ability to remember to perform planned actions in a future moment and it is of fundamental importance for an independent and autonomous lifestyle from development to late adulthood. Deficits in episodic memory and executive functions, which are involved in PM are characteristic features of mild cognitive impairment (MCI) and Alzheimer’s disease (AD). Considering that the number of older adults is drastically increasing over the next decades, it is of great interest to understand how PM decline in healthy older adults and patients with different degree of cognitive decline. The present meta-analysis included 46 studies investigating PM performance in AD patients (17 studies) and people with MCI (24 studies); 5 studies included both clinical conditions in the same article. The 46 studies contributed a total of 63 independent samples and 129 effect sizes from 4668 participants (2115 patients and 2553 controls). Unlike previous reviews of the literature, our results with a larger and updated sample of studies confirmed lower PM abilities in AD compared to MCI and controls, although we did not observe conclusive differences between event-based and time-based PM in patients. Surprisingly, PM deficits shown by MCI and AD patients have decreased across years, in parallel to a reduction of the evidence of publication bias and an increase in the number of observations per task. We propose the use of more reliable research designs as one plausible explanation for the reduction of PM impairments.

**Supplementary Information:**

The online version contains supplementary material available at 10.1007/s11065-023-09626-y.

## Introduction

Cognitive impairments that occur due to mild cognitive impairment (MCI) or more severe forms of dementia have been well described in terms of memory deficits or executive dysfunction (Bastin et al., 2019; Cohen et al., 2019; Glisky, 2007; Mimura & Yano, 2006), with the predominant difficulties in retrospective memory, at least in earlier stages of the Alzheimer’s diseases (AD; Huppert & Beardsall, 1993; Murman, 2015). In the 1990s, increasing interest was dedicated to another form of memory process called prospective memory (PM). PM is defined as remembering to carry out intended actions when a specific target occurs or at an appropriate time in the future (Burgess & Shallice, 1997; Ellis & Kvavilashvili, 2000; Kliegel, Jager, et al., 2008, Kliegel, McDaniel, et al., 2008; McDaniel & Einstein, 2007, 2011). It is a highly complex process that requires formulating plans and intentions, retaining the information, and then executing the planned intention at the appropriate future moment. Two distinct PM components have been identified in the execution of a PM task: the *retrospective component*, which is responsible for the initial encoding and long-term retention of the content of the intention, and the *prospective component*, which refers to the ability to autonomously activate the intention at the right moment without any explicit prompt to recall being given (McDaniel & Einstein, 2000). An additional distinction concerns the cue that triggers the PM action: if the cue is event-based, a person performs a PM action when a specific event occurs; conversely, whereas if the cue is time-based, a person forms a self-generated intention to perform an action at a specific time in the future. Everyday life examples of PM activities concern our ability to remember the appointment with the doctor at 4 pm (i.e. time-based PM) or to remember to buy the milk at the store on your way home (i.e. event-based PM; Kliegel, Jager et al., 2008, Kliegel, McDaniel, et al., 2008).

Executive and declarative memory processes are differently implicated in the two PM components. Indeed, the encoding and long-term retention of the associative relationship between a specific event or time and the concrete actions to be performed requires the correct functioning of the declarative memory system. Conversely, the executive system is mainly implicated in controlling the mental operations needed to spontaneously activate the prospective intention at the appropriate time or at the occurrence of the specific cue. To resemble everyday experiences, laboratory PM tasks are typically embedded in an ongoing task; participants need to share their cognitive resources between performing the ongoing task and keeping track of the PM task. Periodically they must monitor for the occurrence of the appropriate cue or time to initiate task execution. When finally, the appropriate moment occurs (either cue or time triggered), they must stop performing the ongoing task and begin performing the intended action. Top-down attentional control, strategic monitoring of the external environment and/or of the time passing and shifting between concurring activities are all cognitive abilities under the control of the executive system (Laera et al., 2021; Martin et al., 2003; Schnitzspahn et al., 2013) which is known to be affected by age (McFarland & Glisky, 2009).

Previous neuroimaging studies have identified a significant role of anterior frontal regions in PM functions (Burgess et al., 2001, 2003; Lamichhane et al., 2018; Zhuang et al., 2021). Brodmann Area 10 (BA 10) has been demonstrated in directing attention toward either stimulus-oriented or stimulus-independent thoughts (Burgess et al., 2007). The medial and lateral portions of the anterior prefrontal cortex are critical in balancing attention between the external ongoing stimuli and the internally represented PM intention. A recent meta-analysis (Cona et al., 2015) further indicated that PM may rely on the dorsal frontoparietal network which is involved mainly in the maintenance phase and seems to mediate the strategic monitoring processes (top-down attention both towards external stimuli and to internal memory contents). The ventral frontoparietal network is recruited in the retrieval phase and probably the bottom-up attention is captured by external PM cues and activated, internally, by intention stored in memory. Together with other brain regions (i.e. insula and posterior cingulate cortex), the ventral frontoparietal network would support the spontaneous retrieval processes. Neuroimaging studies investigating time-based PM have identified specific activations in the superior and middle prefrontal cortex as well as the precuneus (Gonneaud et al., 2014). Okuda et al. (2007) revealed that the left rostral prefrontal cortex was found to be more active in the time-based compared with the event-based PM tasks but also a bilateral decrease in blood flow in medial BA 10 regions during the event-based relative to the time-based PM tasks. Moreover, the authors found that time-based PM recruited far more prefrontal regions than event-based PM did depending on clock availability. In a recent study, Morand and colleagues (2021) exhibited that reduced time-based PM performance in older adults correlated with diminished white matter integrity, particularly within the tracts of the superior fronto-occipital fasciculus, whereas no correlations with grey matter volume were found.

Deficits in memory and executive functioning, which are involved in PM (Laera et al., 2021; McFarland & Glisky, 2009; Schnitzspahn et al., 2013), are characteristic features of mild cognitive impairment (MCI) and dementia (e.g. Alzheimer’s disease, AD; Arnáiz & Almkvist, 2003; Bäckman et al., 2005; Baddeley et al., 2001; Petersen, 2004). Mild cognitive impairment (MCI) is an intermediate clinical state between normal cognitive decline due to ageing and dementia (Albert et al., 2011; Díaz-Mardomingo et al., 2017). It has been observed that people with MCI progressed to dementia at very different rates, with an average conversion rate of 10% per year; Petersen (2003) reported that after approximately 6 years, 80% of the MCI cohort has progressed to dementia. However, not all individuals with MCI progress to AD (Petersen et al., 1999) raising the concern that MCI is both a clinically and etiologically heterogeneous grouping. On the other hand, AD is a progressive age-related neurodegenerative disease associated with distinct pathological changes (extracellular accumulation of amyloid-beta-containing plaques and intracellular development of tau-containing neurofibrillary tangles) in cortical and subcortical regions (McKhann et al., 2011). According to the amyloid cascade theory, the main cause of AD consists in the precipitation of beta-amyloid proteins and the formation of extracellular plaques, which leads to inflammatory processes and finally results in cognitive deficits (de Vrij et al., 2004; Morishima-Kawashima & Ihara, 2002). The possibility that the AD process may begin years before clinical symptoms is evident (Petersen, 2003). Monitoring cognitive decline and more specifically memory and PM in older adults is fundamental at clinical and experimental level to detect, at the individual level, the first manifestation of more severe cognitive decline.

Our capacity to shape and direct our future behaviour is of fundamental importance in the development, pursuit, and maintenance of an independent and autonomous lifestyle from early childhood to late adulthood. Adequate PM abilities are fundamental for social interaction or normal maintenance, such as remembering your friend’s birthday or remembering to stop at the grocery store on the way home from work or paying bills before the due date. Many other PM tasks are central to health needs, in particular for older adults, such as remembering to take medication and remembering to monitor indexes of physical function (e.g. blood sugar levels; Hering et al., 2018). Given the importance of PM tasks in everyday life and the demographics of an increasingly ageing society, it is important to understand PM performance in healthy and clinical ageing.

In 2012 van den Berg and colleagues conducted a meta-analysis investigating event-based and time-based PM performance in healthy and in patients with different degrees of cognitive decline. The meta-analysis included 14 (7 included AD patients, 4 included MCI patients and 3 included both groups)^1^ and showed no statistical difference between the PM impairment in MCI and AD; both types of patients exhibited large deficits in PM compared to healthy older adults (Cohen’s *d* of −1.62). Those results were surprising considering that AD patients exhibit more severe overall cognitive impairments than MCI patients (Albert et al., 2011; Arnáiz et al., 2003), for what the authors stated that it corroborated earlier suggestions that PM is already affected in the early stages of cognitive decline (Huppert & Beardsall, 1993). In addition, the size of the impairment was comparable for event-based and time-based PM, as well as for PM and RM, and the meta-analysis did not find evidence of publication bias. Those findings were of particular interest bringing light to the importance of including PM measures in clinical settings to further test PM decline in the early stages of dementia.

Since 2012 we have observed an increasing interest in PM performance in the clinical population that mirrors the great interest in understanding PM impairment and developing new training to compensate for and enhance remaining PM competencies (Hering et al 2018; Kliegel et al., 2016). Considering that the number of older adults is drastically increasing over the next decades, it is timely to understand how PM decline as one gets older and signs of cognitive decline more severe. The primary aim of the present meta-analysis was to quantify the nature and extent of PM deficits in MCI and AD incorporating all the new evidence that appeared in the last decade to the literature reviewed in the previous meta-analysis. PM is a valid construct in neuropsychological assessment in patients with MCI and AD; a better understanding of prospective memory abilities in patients with MCI or AD will provide the opportunity to better comprehend the functioning of PM and to assist researchers and clinicians in shaping increasingly effective and patient-centred intervention projects.

## Method

### Literature Search (PRISMA)

**Fig. 1:**
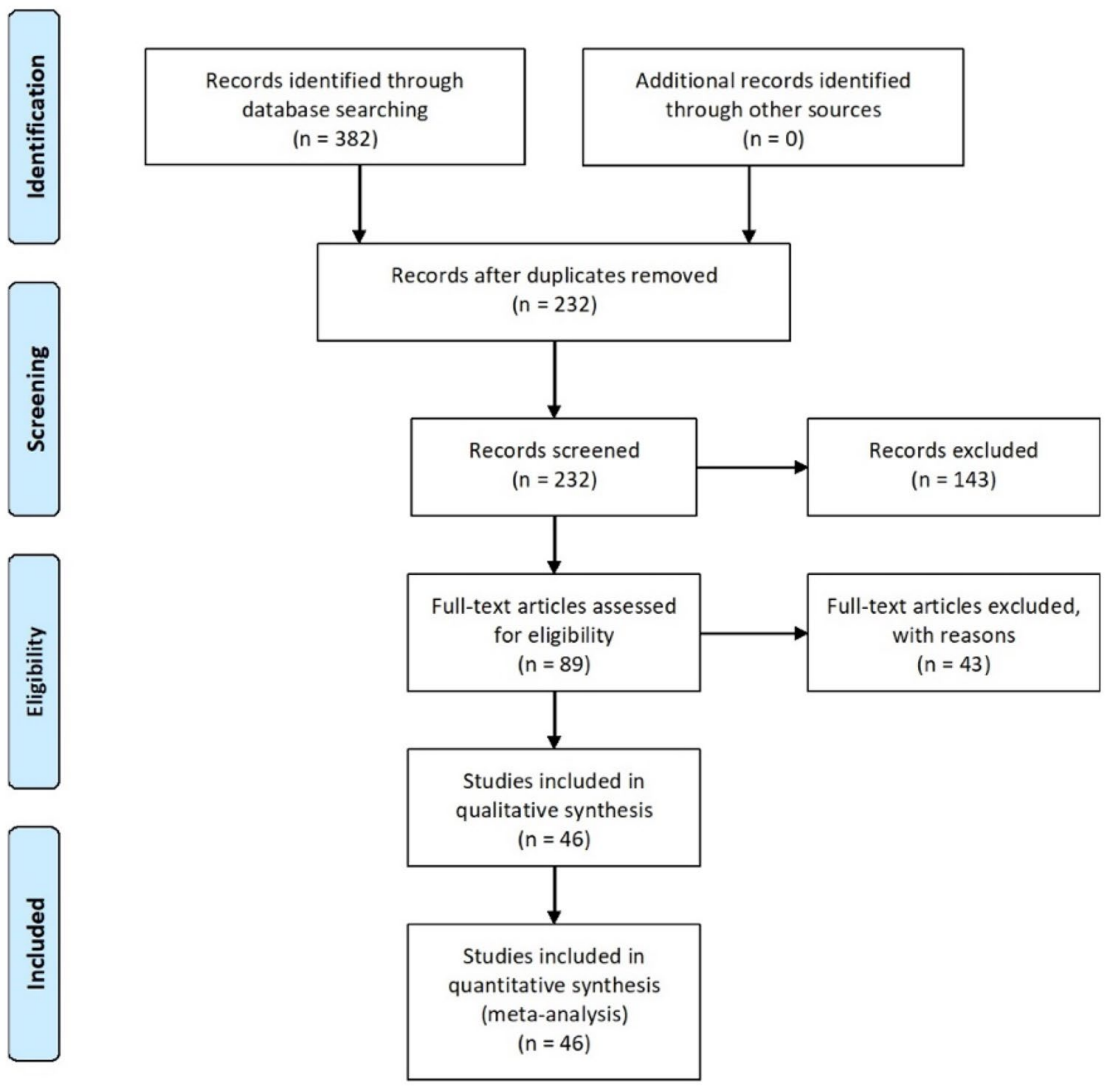
Flowchart of the studies included in the systematic review and meta-analysis

A systematic search strategy was performed following the PRISMA recommendations (Moher et al., 2009; Page et al., 2021). Starting from previous meta-analysis (van den Berg et al., 2012 literature search from 1990 to July 1, 2011), we conducted our search starting from 2011 to February 2022. Firstly, we consulted PsycInfo, PubMed, and Web of Science using the terms: “prospective memory”, “event-based prospective memory”, “time-based prospective memory”, “PM”, “event-based PM”, “time-based PM”, “EBPM”, or “TBPM”, in combination with “Dementia”, “Alzheimer”, “AD”, “mild cognitive impairment” or “MCI”. Reference lists from published reviews, books, and chapters were additionally checked to identify studies that might have been missed by the databases search. The literature search was conducted by the librarian assistant working at the Department of General Psychology, University of Padova; articles selection was conducted independently by GM and RRC and two research assistants at the Department of General Psychology. Any difference was resolved by discussion until a consensus was reached. In total, we found 382 potentially relevant studies and 232 records remained after duplicates were removed. Among them, 46 studies met the inclusion criteria described below and formed the sample for our meta-analysis (Fig. 1).

### Inclusion Criteria

The inclusion criteria were as follows:

1. Published articles or theses reporting measures of event-based or time-based PM abilities in patients with AD or MCI,
2. The studies additionally assessed PM in a group of healthy older participants used as a comparison group,
3. Designs with standard encoding of the instructions, excluding conditions in which the participants received strategies that presumably affect PM performance (such as implementation intention encoding; Lee et al., 2016; Shelton et al., 2016),
4. The studies contained sufficient information to calculate at least one effect size (otherwise, authors were contacted, and the studies included if the information was provided),
5. Participants did not suffer from any other neurological or psychiatric condition.

### Statistical Analysis

#### Effect Size

As the studies used different tasks of PM, we opted for the standardized mean difference as an estimator of the effect size of accuracy in PM tasks (for the sake of homogeneity between the studies; measures of RTs were not included). Specifically, we used the between-group Hedges’ *g*, which reduces the bias of small samples in classic Cohen’s *d* through a correction factor (*J*),

1 $$g=J\times \frac{{\text{M}}_{\text{patient}} \, - \, {\text{M}}_{\text{control}}}{{\text{SD}}_{\text{pooled}}},$$

with variance,

2 $${V}_{g}={J}^{2}\times \frac{{n}_{patient}+{n}_{control}}{{n}_{patient}\times {n}_{control}}+\frac{{d}^{2}}{2\times \left({n}_{patient}+{n}_{control}\right)},$$

where *M*_patient_ and *M*_control_ represent the means of AD/MCI patients and healthy controls, respectively; *n*_patient_ and *n*_control_ are the number of participants in each group; and *SD*_pooled_ is the pooled standard deviation for the scores of both groups (Borenstein et al., 2021). In those studies, in which the size of the control group exceeded 1.5 times the size of the group of patients, the sampling variance was calculated by replacing the number of control participants by the number of patients:

3 $${V}_{g}={J}^{2}\times \frac{2\times {n}_{patient}}{{n}_{patient}^{2}}+\frac{{d}^{2}}{4\times {n}_{patient}},$$

With the previous procedure, we prevent some large studies, mainly because of the large size of their control sample, from contributing more (i.e. smaller variance) to the final meta-analytic effect than other smaller studies but with similar size of their group of patients. Moreover, *J* was calculated as follows:

4 $$J=1-\frac{3}{4\times \left({n}_{patient}+{n}_{control}-2\right)-1}$$

Negative values of *g* represent worse PM performance for patients than controls, whereas positive values index the opposite case. We multiplied by –1 to maintain this coherence among the effects (of note, this procedure was only applied to the outcomes in Shelton et al., 2016).

#### Meta-analytic Approach, Heterogeneity, and Moderator Analysis

Due to most of the included studies contributed with more than one effect size from the same sample, we used the robust variance estimation method (RVE; Hedges et al., 2010) using the *robumeta* package for R (Fisher et al., 2017) to conduct multilevel models, with a prespecified within-study effect-size correlation of .80 (although sensitivity analyses were conducted with other correlation values to test the robustness of the models: 0, .2, .4, .6, and 1). The significance level was set at .05. This method allows for dealing with a correlated structure of outcomes from the same study. We chose a correlated dependence model with small-sample corrections (Tipton, 2015) and effect sizes were nested within each independent sample of participants. Note that some studies also contributed with several experiments and/or multiple samples of patients, so we decided to select independent samples as a nesting variable (i.e. the main source of dependency). First, we tested the overall difference in PM between AD/MCI patients and healthy controls. Moreover, we computed the common heterogeneity indexes: τ^2^ and *I*^2^.

In a second step, we repeated the analyses including variables that could have a moderating effect on the final estimate and possibly account for part of the heterogeneity. We, thus, fitted separate multilevel meta-regressive models with the following moderators, one model per moderator:

1. Neurological condition: AD patients vs. controls or MCI patients vs. controls;
2. Mean Mini-Mental State Examination (MMSE) score of the group of patients^2^;
3. The standardized mean difference (Hedges’ *g*) in the reported neuropsychological tests of (3a) retrospective memory, (3b) executive functions, (3c) working memory, and (3d) processing speed^3^;
4. Mean age of the study participants (in years);
5. Mean years of education of the study participants;
6. Type of measure regarding its cue for action: event-based or time-based PM;
7. Type of PM task: classic neuropsychological PM tasks or other PM tasks^4^;
8. Year of publication of the study;
9. If the study was published before or after the meta-analysis by van den Berg et al. (2012).

Moreover, we conducted a meta-regression with the standard error of the effect size as a covariate to test for the existence of publication bias. If the publication process favours significant results that confirm the predominant theories than null outcomes, it would be more likely to observe larger effects in smaller studies (i.e. small-study effect). It could be translated to asymmetrical distributions of the effect sizes, especially within studies with larger standard error, with few small-to-null results (Egger et al., 1997). Therefore, a way to test the existence of publication bias is through a meta-regressive model using standard error as a predictor. Moreover, the intercept of that meta-regression can be used as the adjusted overall effect (i.e. the intercept when the standard error is close to zero; Stanley & Doucouliagos, 2014). In the present work, we chose a variance-stabilizing transformation for the standardized mean difference (*h*) to conduct the test of asymmetry this transformation prevents the artefactual dependence between the effect size and its precision estimate (Pustejovsky & Rodgers, 2019). In parallel, we implemented the same analysis with the ordinary Hedges’ *g* and a modified formula of the sampling variance (*W*) to adjust the final effect without changing the scale of the effect size, unlike the variance-stabilizing transformation.

Finally, to find out which combination of moderators provided the best fit for the data, we carried out a backward stepwise selection (α_exclusion_ = .10) with all the moderators. This procedure would consider more complex structures of moderators and look into the residual heterogeneity of the best meta-regressive model.

## Results

The meta-analysis included 46^5^ studies investigating the differences in PM in AD patients compared to healthy older adults (17 studies), in people with MCI (24 studies), or both conditions in the same article (5 studies: Kazui et al., 2005; Massa et al., 2020; Thompson et al., 2010; Thompson et al., 2011; Troyer & Murphy, 2007; Tables 1 and 2). In three samples (Huppert & Beardsall, 1993; Mori & Sugimura, 2007; Thompson et al., 2010, 2011) AD patients were mixed with patients with other types of dementia (e.g. Lewy-body or vascular dementia), but the latter represented a small proportion of the samples (Huppert & Beardsall: 6%; Thompson et al.: 10%). All the findings in the present work remained identical when these three samples were excluded in subsequent sensitivity analyses. The use of different within-study effect-size correlations in RVE models (i.e. 0, .2, .4, .6, and 1, instead of the prespecified .8) also did not affect the results. The 46 studies contributed a total of 63 independent samples and 129 effect sizes from 4668 participants (2115 patients and 2553 controls).

**Table 1:** Characteristics of studies including AD patients

						**AD patients**					**Control group**				
**Study (year)**	**Independent samples (in the same article)**	**Included in van den **Berg et al. (2012)	**PM type**	***PM task description***	***k***	***n***	**Age (years)**	**Sex (M:F)**	**Education (years)**	**MMSE**	***n***	**Age (years)**	**Sex (M:F)**	**Education (years)**	**MMSE**
Dermody et al. (2016)		No	TB and EB	Modified version of CAMPROMT (3 TB cues and 3 EB cues)	2	12	63.3	7:5	12.6	-^a^	12	69.0	6:6	13.6	-^a^
Duchek et al. (2006)		Yes	EB	Respond to target word in general knowledge test (8 EB cues)	2	26	77.7	-	14.3	-^b^	36	80.9	-	15.0	-^b^
El Haj et al. (2018)		No	EB	Respond to target word while reading short tests (12 EB cues)	2	24	71.6	7:17	8.8	21.38 (1.81)	27	68.9	10:17	9.2	27.74 (1.48)
Farina et al. (2013)	Younger AD	No	EB	Mark the occurrence of all number 7 cards during a computerized card sort task (2 decks, 8 EB cues)	1	34	74.7	11:23	-	23.53 (3.27)	42	72.6	24:18	-	29.34 (0.83)
	Older AD		EB		1	45	84.8	21:24	-	24.33 (3.19)	42	72.6	24:18	-	29.34 (0.83)
Gao et al. (2013)		No	EB	Arrow and colour-bar PM task (14 EB cues)	1	26	76.0	8:18	3.8	21.2 (-)	40	74.8	17:23		28.1 (-)
Huppert and Beardsall (1993)	Minimal dementia	Yes	EB	RBMT total PM score	4	12	87.3	5:7	14.5	19.8 (-)	27	81.1	10:17	14.5	24.8 (-)
	Mild/moderate dementia		EB		4	9	80.6	3:6	14.1	15.3 (-)	27	81.1	10:17	14.5	24.8 (-)
Jones et al. (2006)		Yes	EB	Remind the test leader to make a phone call (1 cue)	1	46	84.0	2:44	8.2	24.43 (2.83)	188	84.0	43:145	8.9	27.06 (2.18)
Kamminga et al. (2014)		No	TB and EB	CBPMT (3 EB cues and 3 TB cues)	2	8	62.9	6:2	12.8	-^a^	11	70.0	-	13.9	-^a^
Kazui et al. (2005)		Yes	EB	RBMT (Belonging; Appointment; Message (immediate and delayed))	4	48	67.7	18:30	11.4	21.9 (2.3)	48	66.7	18:30	11.5	28.2 (1.8)
Kinsella et al. (2007)	Study 1	Yes	EB	Respond to target word during Text-Reading Task (12 EB cues)	1	14	79.1	5:9	11	23.21 (3.28)	14	75.7	5:9	11.6	28.89 (1.46)
	Study 2		EB		1	16	80.5	5:11	10.9	23.25 (2.96)	16	79.3	5:11	11.6	28.75 (1.73)
Lecouvey et al. (2019)		No	TB and EB	Walk in a virtual town (6 EB cues; 1 TB cue)	3	17	79.3	7:10	9.7	22.82 (2.83)	15	76.5	5:10	12.3	28.80 (1.21)
Lee et al. (2016)		No	EB	Category decision task with focal and non-focal EB cues	2	19	78.6	-	15.3	27.2 (0.49)	17	74.8	-	14.9	28.8 (0.43)
Martins and Damasceno (2008)		Yes	EB	RBMT (Belonging; Appointment) two EB tasks developed by authors: Animals’ test and Clock test	1	20	75.6	9:11	5.6	22.6 (1.9)	20	74.1	9:11	5.8	29.0 (1.3)
Massa et al. (2020)^c^		No	TB and EB	General knowledge questions (ongoing); TB = call the examiner every 5 min (5 TB cues); EB = respond to target words (5 EB cues)	2	18	74	10:8	11.7	27.7 (1.6)	23	71.1	11:12	11.8	29.0 (1.1)
Maylor et al. (2002)	Experiment 1	Yes	TB & EB	Watch a movie (ongoing task) EB = respond to animals; TB = press after 3 min	2	24^d^	68.5	10:14	10.1	22.1 (3.6)	30^d^	67.3	12:18	12.3	-
	Experiment 2		EB		2	18^d^	68.7	5:13	10.6	20.9 (3.8)	20^d^	68.1	6:14	12.0	-
Mori and Sugimura (2007)		Yes	EB	RBMT total PM score	1	52	81.2	0:52	9.1	17.6 (4.1)	50	80.0	0:50	8.9	27.2 (2.2)
Shelton et al. (2016)		No	TB and EB	Virtual Week (2 trial days, 2 EB cues and 2 TB cues per day)	1	17	78.6	6:11	14.9	26.50 (3.2)	19	74.8	8:11	14.7	28.80 (1.3)
Thompson et al. (2010)		Yes	TB and EB	Virtual Week (2 trial days, 2 trial days, 2 EB cues and 2 TB cues per day)	1	39	79.8	20:19	12.0	25.3 (4.30)	53	77.8	22:31	11.3	28.7 (1.42)
Thompson et al. (2011)		No	TB and EB	Naturalistic task executed over 2 days; TB = turn-on the device, EB = respond to the question	1	22	79.1	11:11	11.1	26.8 (2.53)	45	77.7	17:28	11.5	28.7 (1.34)
Troyer and Murphy (2007)		Yes	TB and EB	Cognitive testing (ongoing); TB = Report time every 30 min (4 targets); EB = Use coloured pen in task requiring writing (4 targets)	2	24	78.4	10:14	12.5	25.5 (2.2)	42	75.1	25:17	13.8	28.7 (1.2)
Tse et al. (2015)	Early-stage AD	No	EB	Arrow and colour-bar PM task (24 EB cues)	1	125	78.7	56:69	4.7	24.79 (2.94)	125	75.1	63:62	7.1	27.72 (1.84)
	Mild AD		EB		1	30	80.2	9:21	4.1	21.43 (.47)	125	75.1	63:62	7.1	27.72 (1.84)
Zhuang et al. (2021)		No	TB and EB	Semantic categorization task (ongoing); EB = respond to target word (13 cues); TB = press every 30 s (13 cues)	2	22	75.3	11:11	8	22.27 (4.03)	31	67.3	17:14	10	28.19 (1.70)

*EB* event-based, *TB* time-based, *MMSE* Mini Mental State Examination, *CAMPROMT* Cambridge Prospective Memory Test, *RBMT* Rivermead Behavioural Memory Test, *CBPMT* Cambridge Behaviour Prospective Memory Test

^a^Global state of cognitive functioning measured with the Addenbrooke’s Cognitive Examination Revised

^b^Global state of cognitive functioning measured with the Information subscale of the Wechsler Adult Intelligence

^c^AD and MCI combined in the same group of patients

^d^Patients and controls were divided into two groups, one half of the participants with an event-based measure of PM and the other half with a time-based measure

**Table 2:** Characteristics of studies including MCI patients

						MCI patients					Control group				
**Study (year)**	**Independent samples (in the same article)**	**Included in van den Berg et al.** (2012)	**PM type**	***PM task description***	***k***	***n***	**Age (years)**	**Sex (M:F)**	**Education (years)**	**MMSE**	***n***	**Age (years)**	**Sex (M:F)**	**Education (years)**	**MMSE**
Aronov et al. (2015)		No	TB and EB	RPA-ProMem (2 EB cues and 2 TB cues)	1	52	80.5^a^	17:35^b^	14.5^a^	-	91	80.5^a^	29:62^b^	14.5^a^	-
Beaver and Schmitter-Edgecombe (2017)		No	EB	To remember to record an activity	1	37	72.6	-	14.9	-	133	71.7	-	16.4	-
Belmar et al. (2020)		No	TB and EB	MIST (4 EB cues and 4 TB cues)	2	41	69.2	15:26	-	28.2 (1.8)	40	66.3	17:23	-	29.1 (0.9)
Blanco-Campal et al. (2009)		Yes	EB	Lexical decision task (10 specific targets and 10 non-specific cues)	4	19	71.1	9:10	-	25.72 (1.97)	21	72.5	6:15	-	29.4 (0.7)
Cheng et al. (2021)		No	EB	Modified Six-Elements Task	2	15	65.0	10:18	9.4	27.00 (2.24)	15	63.00	10:22	10.4	28.16 (1.39)
Chi et al. (2014)	aMCI	No	EB	Word-categorization (ongoing); 4 focal and 4 non-focal EB cues	2	15	83.1	6:9	14.6	-	98	81.4	36:62	14.8	-
	naMCI		EB		2	18	81.2	3:15	12.2	-	98	81.4	36:62	14.8	-
Costa et al. (2010)		Yes	TB and EB	Paper and pencil exercises (ongoing); 3 EB cues and 3 TB cues	3	20^c^	72.2^c^	8:12^c^	10.2^c^	26.0 (1.4)^c^	20	71.5	11:9	10.5	28.2 (1.4)
Costa et al. (2011)		No	EB	Respond to target word in bisyllabic words test (10 EB cues)	4	24	72.7	14:10	9.4	26.3 (1.3)	24	70.9	14:10	10.2	28.9 (1.3)
Costa et al. (2015)	Single domain aMCI	No	TB	Neuropsychological tests (ongoing); 4 TB cues	1	16	69.4	10:6	13.0	26.2 (2.3)	43	68.8	17:26	12.4	27.7 (1.5)
	Multiple domain aMCI		TB		1	13	72.0	8:5	11.8	26.2 (1.8)	43	68.8	17:26	12.4	27.7 (1.5)
Crook-Rumsey et al. (2022)		No	EB	Respond to target word in 1-back word categorization task (60 EB cues)	6	39	72.9	15:24	-	-	27	77.5	15:12	-	-
Delprado et al. (2012)		No	TB and EB	CAMPROMPT (3 EB cues and 3 TB cues); 2 single EB tasks (prompt card and envelope)	4	84	74.9	37:47	13.0	27.18 (1.79)	84	74.8	37:47	13.3	28.86 (0.93)
Karantzoulis et al. (2009)		Yes	TB and EB	MIST (4 EB cues and 4 TB cues)	3	27	75.7	12:15	13.0	-	27	73.0	7:20	14.2	-
Kazui et al. (2005)		Yes	EB	RBMT [Belonging; Appointment; Message (immediate and delayed)]	4	24	66.9	9:15	11.5	26.7 (1.9)	48	66.7	18:30	11.5	28.2 (1.8)
Kinsella et al. (2016)		No	TB and EB	CAMPROMPT (3 EB cues and 3 TB cues)	1	106	76.1	45:61	-	26.95 (1.87)	113	72.3	31:82	-	28.93 (1.01)
Lajeunesse et al. (2021)		No	TB and EB	Ecological Test of Prospective Memory	2	25	74.7	9:16	14.1	-^d^	25	71.9	8:17	15.7	-^d^
Lajeunesse et al. (2022)	Training group	No	TB and EB	Ecological Test of Prospective Memory	2	12	73.8	4:8	14.7	-^d^	12	72.0	4:8	15.0	-^d^
	No-training group		TB and EB		2	12	76.3	5:7	13.9	-^d^	12	71.7	4:8	16.8	-^d^
Massa et al. (2020)^e^		No	TB and EB	General knowledge questions (ongoing); TB = call the examiner every 5 min (5 TB cues); EB = respond to target words (5 EB cues)	2	18	74	10:8	11.7	27.7 (1.6)	23	71.1	11:12	11.8	29.0 (1.1)
Niedzwienska et al. (2017)	Focal cue	No	EB	Colour photographs (focal and non-focal EB cues)	1	12	79.8	7:10	11.5	27.59 (1.77)	24	76.4	10:14	12.6	29.54 (0.78)
	Non-focal cue		EB		1	17	77.7	6:11	11.4	27.41 (1.87)	22	76.4	8:14	12.5	29.32 (0.89)
Pereira et al. (2015)		No	EB	Words categorization task (12 EB cues)	1	64	73.0	-	9.2	25.84 (3.51)	64	69.7	-	10.9	29.02 (0.85)
Pereira et al. (2018)		No	EB	Respond to target word in 1back word task	1	32	76.8	-	14.4	-	32	76.1	-	13.6	-
Rabin et al. (2014)	aMCI	No	TB and EB	RPA-ProMem (2 EB cues and 2 TB cues); MIST (4 EB cues and 4 TB cues)	3	18	81.6	7:11	13.6	-	118	80.3	40:78	15.0	-
	naMCI		TB and EB		3	38	80.4	7:31	11.8	-	118	80.3	40:78	15.0	-
Schmitter-Edgecombe et al. (2009)	aMCI	Yes	EB	During cognitive testing: Ask examiner for pill and bottle after every task	1	27	71.3	13:14	16.1	26.85 (-)	42	72.5	17:25	16.1	28.71 (-)
	naMCI		EB		1	15	72.2	4:11	15.9	27.40 (-)	42	72.5	17:25	16.1	28.71 (-)
Tam and Schmitter-Edgecombe (2013)		No	EB	Respond to target word in working memory task	1	24	73.9	12:12	16.2	27.22 (1.65)	24	73.3	9:15	16.1	28.63 (1.38)
Thompson et al. (2010)		Yes	TB and EB	Virtual Week (2 trial days, 4 cues per day)	1	48	78.6	26:22	12.2	28.0 (1.56)	53	77.8	22:31	11.3	28.7 (1.42)
Thompson et al. (2011)		No	TB	Naturalistic task executed over 2 days; TB = turn-on the device, EB = respond to the question	1	31	78.8	18:13	11.2	27.7 (1.50)	45	77.7	17:28	11.5	28.7 (1.34)
Thompson et al. (2017)		No	EB	Brief Smell Identification test (3 salient and 3 non salient EB cues)	1	236	80.2	124:112	11.3	27.80 (1.58)	421	80.0	167:254	11.9	28.72 (1.32)
Troyer and Murphy (2007)		Yes	TB and EB	Report time every 30 min (3 TB cues) and use a pen (3 EB cues) during the clinical interview and neuropsychological assessment	2	45	75.8	24:21	13.6	27.8 (1.4)	42	75.1	25:17	13.8	28.7 (1.2)
Wang et al. (2012)	aMCI	No	TB and EB	2 TB cues; 1 EB cue	3	133	65.5	62:71	12.6	27.30 (1.80)	122	63.6	50:72	12.5	28.24 (1.74)
	naMCI		TB and EB		3	72	63.3	32:40	12.0	27.49 (1.88)	122	63.6	50:72	12.5	28.24 (1.74)
Zhou et al. (2012)		No	TB and EB	Various cognitive measure (ongoing); 2 TB cues and 2 EB cues	3	19	73.4	10:9	12.0	26.82 (2.04)	22	70.5	9:13	13.6	27.67 (2.01)

*aMCI* amnesic mild cognitive impairment, *naMCI* non-amnesic mild cognitive impairment, *EB* event-based, *TB* time-based, *MMSE* Mini-Mental State Examination, *RPA-ProMem* Royal Prince Alfred Prospective Memory Test, *MIST* The Memory for Intentions Test

^a^Values from the whole sample, not individualized per group

^b^Estimated values according to the proportion of women in the whole sample (68.2%)

^c^Values from the whole sample of MCI patients, combining aMCI and naMCI

^d^Global state of cognitive functioning measured with the Montreal Cognitive Assessment

^e^AD and MCI combined in the same group of patients

Consistent with the findings in the preceding literature, patients with AD and MCI showed remarkable impairments in PM compared to healthy controls, *g* =  −1.12 [−1.27, −0.98], *p* < .0001. Contrasting with the meta-analysis by van den Berg et al., 2012, this result arose from a pool of effect sizes that were highly variable among themselves, more than could be explained by sampling error (i.e. heterogeneity): τ^2^ = 0.24, *I*^2^ = 77.86%. It suggests that a great portion of the observed variability between the effect sizes of the studies (77.86%) was potentially due to the influence of moderating variables and other sources of variability different from chance. Studentized residuals (> 2) and Cook’s distance [> 4/(*n* − 1)] allowed us to identify one outlier study contributing with disparate outcomes (*g* <  −3.4; Dermody et al., 2016), probably because of its small sample of AD patients (12 participants). Another reason for those outlying effects would be that the necessary information for estimating them was not available in the manuscript, and we extracted it from the graphs instead (using WebPlotDigitizer, https://automeris.io/WebPlotDigitizer). After excluding the outlying outcomes from Dermody et al. (2016), the overall effect and heterogeneity were reduced, *g* =  −1.1 [−1.24, −0.96], *p* < .0001, τ^2^ = 0.22, *I*^2^ = 76.47%, although heterogeneity remained substantial.

**Fig. 2:**
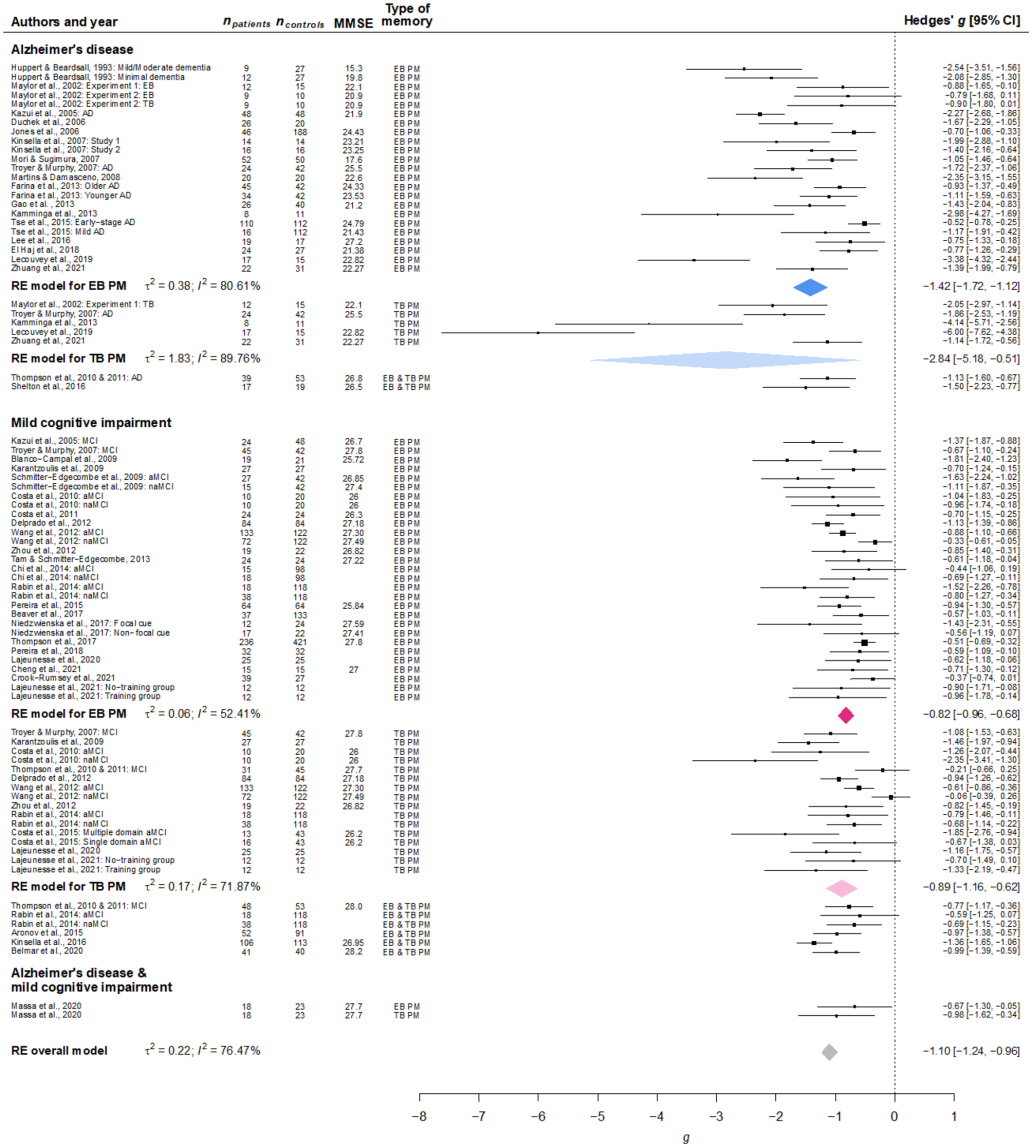
Forest plot of the included. The PM measures within the same type (event-based or time-based) and within the same sample of participants were averaged for depicting purposes. Outlying studies were removed from the plot. aMCI, amnesic mild cognitive impairment; EB, event-based; TB, time-based; MMSE, Mini Mental State Examination; naMCI, non-amnesic mild cognitive impairment. Studies are reported and sorted by year of publication (within each cluster)

The difference between AD and MCI explained part of the observed variability among studies, where AD patients exhibited significantly lower PM performance than patients with MCI (*g* =  −1.45 vs. MCI: *g* =  −0.89; Table 3 and Fig. 2). However, this approach contrasted samples of patients assessed in separate studies and under potentially diverse conditions (different PM tasks, settings, degree of cognitive impairment, etc.). Consistent with the previous result, the difference between AD and MCI patients was statistically significant when the meta-analytic model was fitted only with studies that included samples of both neurological conditions (i.e. assessed under the same procedure), *g*_AD vs. MCI_ =  −0.71 [−0.94, −0.49], *p* = .005, τ^2^ = 0, *I*^2^ = 0%. Similarly, AD patients showed larger PM impairment compared to MCI patients when the model only included classic (and the most established) neuropsychological PM tests (i.e. Cambridge Prospective Memory Test, CAMPROMPT, Wilson et al., 2005; Memory for Intentions Screening Test, MIST, Raskin, 2009; Rivermead Behavioral Memory Test, RBMT, Wilson et al., 1989; and Royal Prince Alfred Prospective Memory Test, RPA-ProMem, Radford et al., 2011), *g* =  −2.08 [−3.02, −1.13], *p* = .003, τ^2^ = 0.20, *I*^2^ = 74.18%. As expected, lower MMSE scores predicted larger impairments in PM (MMSE: *p* = .014). However, the average performance in retrospective memory, executive functions, working memory, and processing speed tests, as well as age and education did not explain PM impairments (*p*s > .05; Supplementary Table 1, 2 include studies reporting the cognitive tests used in each study). There was no difference between time-based and event-based PM measures (*p* = .467), neither when we examined it separately in each neurological condition (AD:* p* = .126; MCI:* p* = .721). It is important to notice that the available number of time-based PM measures, especially in AD patients, remains more limited than for event-based measures (*k* = 25 vs. *k* = 92; AD: *k* = 5 vs. *k* = 37). When the role of this moderator was examined with a multilevel Bayesian meta-analysis,^6^ while the model in MCI patients suggests there was strong evidence against a difference between both types of PM measures (β = 0.05, 95% CrI [−0.10, 0.20], *BF*_10_ = 0.10), the evidence in AD patients is still inconclusive and coherent with larger impairment in time-based PM tasks (β =  −0.41, 95% CrI [−0.92, 0.11], *BF*_10_ = 0.87). The meta-analytic results remained similar when the sample of studies was constrained only to those including both event-based and time-based PM measures (MCI: β = 0.06, 95% CrI [−0.10, 0.22], *BF*_10_ = 0.11; AD: β =  −0.29, 95% CrI [−0.83, 0.25], *BF*_10_ = 0.50). Finally, PM impairments were larger when they were measured with classic neuropsychological tests, *p* = .024.

**Table 3:** Results of moderator analyses

Moderator	β	*t*	*df*	*p*	
Neurological condition^a^	0.51 [0.21, 0,81]	3.39	48.70	.001	AD: *g* = −1.45 [−1.75, −1.16], *k* = 45 MCI: g = −0.89 [−1.02, −0.75], *k* = 80
MMSE	0.26 [0.06, 0.46]	2.81	13.70	.014	*k* = 95
Retrospective memory	0.32 [−0.03, 0.67]	2.06	9.96	.067	*k* = 97
Executive functions	0.17 [−0.30, 0.64]	0.80	9.51	.444	*k* = 71
Working memory	0.22 [−0.15, 0.59]	1.53	5.06	.186	*k* = 57
Processing speed	0.26 [−2.19, 2.71]	0.39	2.37	.729	*k* = 31
Age	−0.01 [−0.04, 0.02]	−0.84	4.59	.444	*k* = 127
Education	0.02 [−0.02, 0.06]	2.82	1.75	.123	*k* = 112
Type of PM measure^b^	−0.14 [−0.52, 0.25]	−0.74	25.20	.467	Event-based: *g* = −1.06 [−1.21, −0.91], *k* = 92 Time-based: *g* = −1.23 [−1.61, −0.84], *k* = 25
Type of PM measure in AD patients^b^	−1.04 [−2.54, 0.46]	−1.95	3.88	.126 (*BF*_10_ = 0.87)	AD event-based: *g* = −1.42 [−1.72, −1.12], *k* = 37 AD time-based: *g* = −2.84 [−5.18, −0.51], *k* = 5
Type of PM measure in MCI patients^b^	−0.06 [−0.42, 0.30]	−0.36	18.70	.721 (*BF*_10_ = 0.10)	MCI event-based: *g* = −0.82 [−0.96, −0.68], *k* = 54 MCI time-based: *g* = −0.89 [−1.16, −0.62], *k* = 19
Classic neuropsychological PM tasks (vs. other PM tasks)	−0.43 [0.06, 0.79]	−2.46	19.50	.024	Classic PM tasks: *g* = −1.44 [−1.80, −1.08], *k* = 37 Other PM tasks: g = −0.98 [−1.13, −0.84], *k* = 90
Year of publication	0.04 [0.01, 0.06]	3.40	21.00	.015	*k* = 127
Published after van den Berg et al.^c^	0.30 [0.02, 0.58]	2.15	54.20	.036	Before: *g* = −1.28 [−1.52, −1.05], *k* = 67 After: *g* = −0.95 [−1.10, −0.79], *k* = 60
*SE*_h_ (small-study bias with a variance-stabilizing transformation)	−2.85 [−4.35, −1.35]	−3.92	25.50	.001	*k* = 127
$\sqrt{W}$(small-study bias)	−2.27 [−3.77, −0.76]	−3.10	24.70	.005	*k* = 127
$\sqrt{W}$× Year of publication	0.08 [0.01, 0.15]	2.36	14.90	.033	*k* = 127
$\sqrt{W}$× Neurological condition^a^	1.55 [0.60, 2.50]	3.29	47.20	.002	*k* = 125
Best meta-regressive model (via backward stepwise selection)					
Neurological condition	0.44 [0.18, 0.71]	3.41	37.50	.002	
Type of PM measure	0.57 [0.24, 0.91]	3.67	13.40	.003	
$\sqrt{W}$(small-study bias)	−2.48 [−3.84, −1.11]	−3.78	20.20	.001	

*k* is the number of effect sizes, *MMSE* Mini-Mental State Examination

^a^The outcomes from Massa et al. (2020), two in total, were not included in the present analysis as they combined AD and MCI patients in the same group

^b^PM scores that do not distinguish between event-based and time-based, such as total scores, were not included in the present analysis

^c^Dummy variable with 0 for studies published before the publication date of van den Berg et al. (2012) and 1 for studies published after

**Fig. 3:**
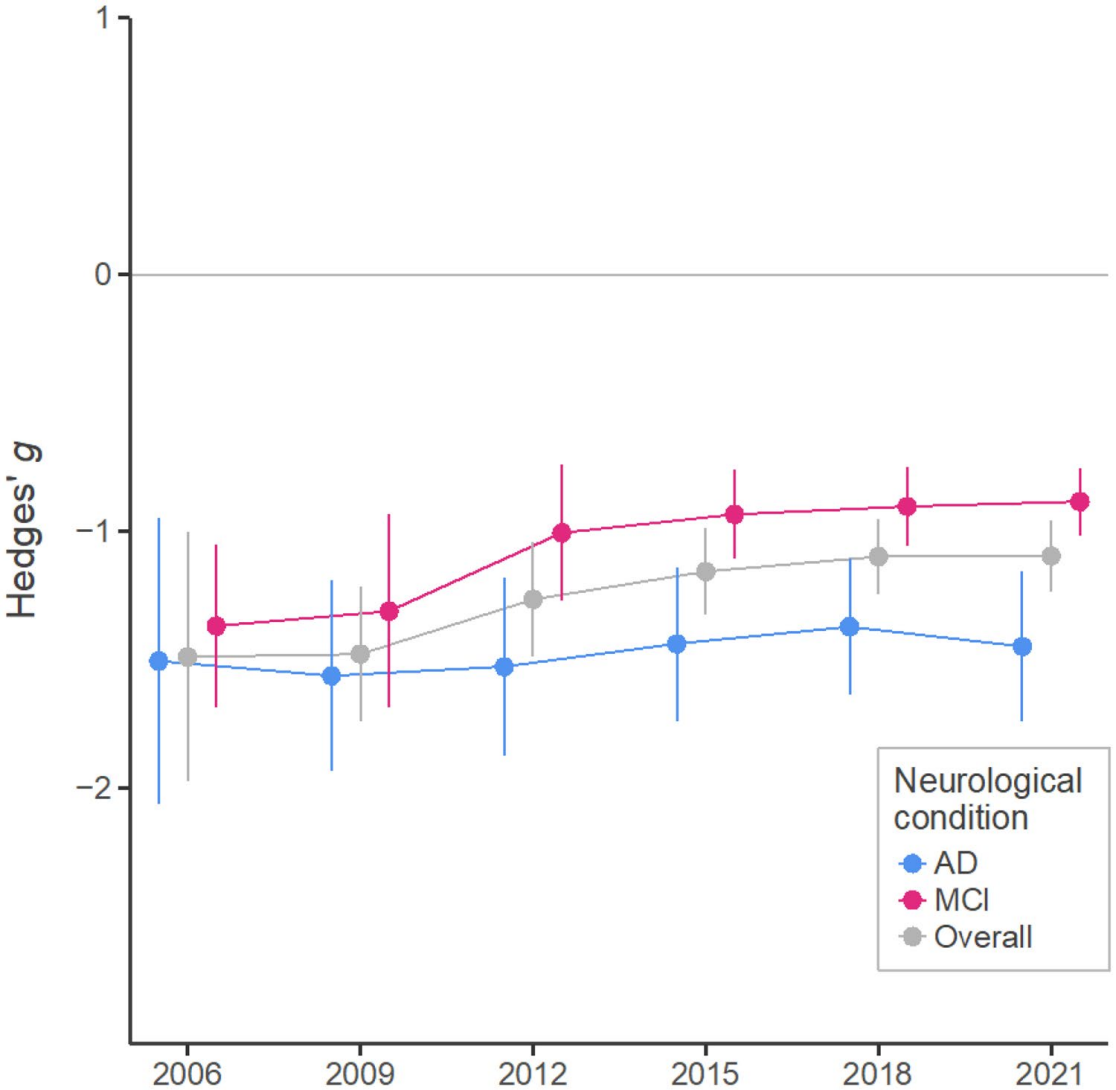
Cumulative meta-analysis across years. Each effect size represents the meta-analytic results of all the included studies that were available by that year (i.e. all studies accumulated up to that period). Whereas the PM impairments for AD patients have remained similar throughout all these years (a reduction of 9%, −1.38/ −1.51), the estimated impairments for MCI patients have been reduced by a third (−0.89/ −1.31)

Interestingly, the year in which the articles were published and if they were published after the meta-analysis by van den Berg et al., both predicted a reduction in the overall effect size (Table 3). As studies have been accumulating in the literature, the estimated overall impairment has been reduced (from *g* =  −1.49 in 2006 to −1.10 in the present; Fig. 3). The reduction has been more remarkable in the case of studies about MCI (from −1.31 to −0.89). In fact, as it was reported by van den Berg et al., the difference between AD and MCI patients in the magnitude of PM impairments was not statistically significant by the date in which the literature search of the previous meta-analysis was limited (July 1, 2011), *p* = .108 (AD: *g* =  −1.53 [−1.87, −1.18]; vs. MCI: *g* =  −1.16 [−1.48, −0.84]).

**Fig. 4:**
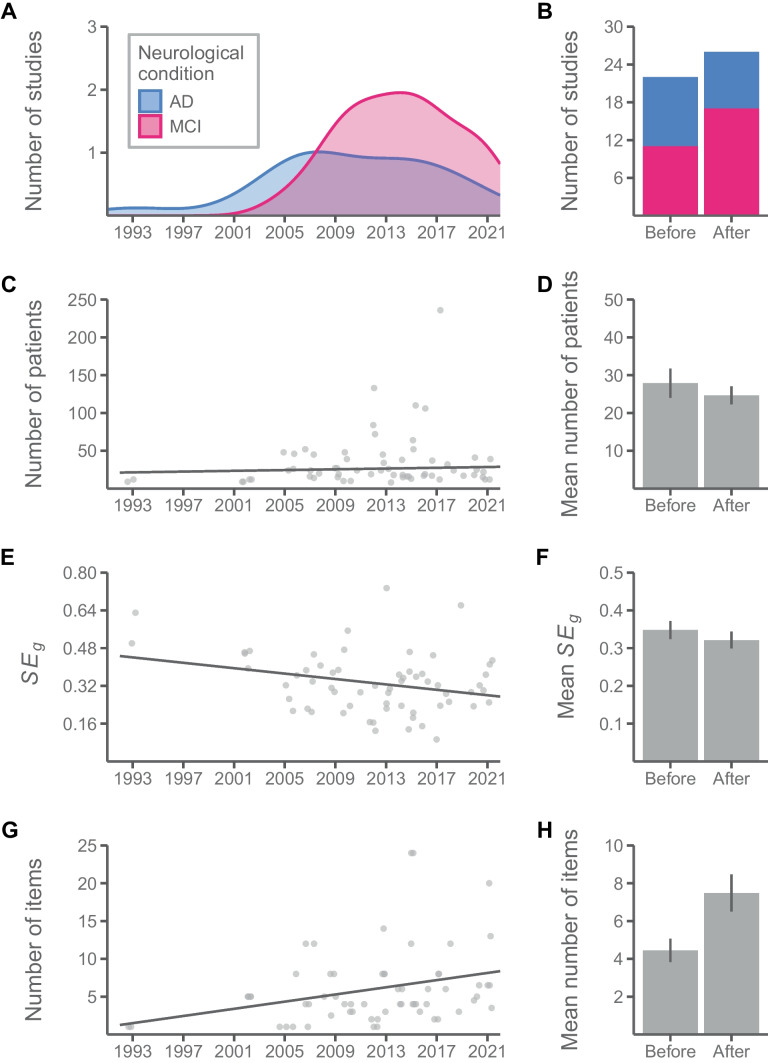
Chronological evolution of **A** the number of published studies investigating PM in AD and MCI, as well as **C** the number of patients, **E** the standard error, and **G** the number of task items in the included studies. Across years, **B** the proportion of studies with MCI patients and (H) the number of task items have increased in the period after the publication of the meta-analysis by van den Berg et al. (2012), while **F** the standard error of the studies has decreased. The size of the samples of patients remained similar across years, and **D** it was comparable before and after the reference time point, especially when removing three studies with unusually large samples (*n* > 100)

One reason for the unexpected reduction in the overall effect size could be the increasing number of articles with MCI patients across years, *r* = .42 [.18, .61], *p* < .001 (Fig. 4A). The proportion of studies investigating PM in MCI patients was smaller before the publication of the meta-analysis than after (46% vs. 67%; Fig. 4B). Given that MCI patients showed smaller PM impairments compared to AD, their greater representation in the latter period may have led to the meta-analytic result being closer to the outcome of MCI patients (Fig. 3).

**Fig. 5:**
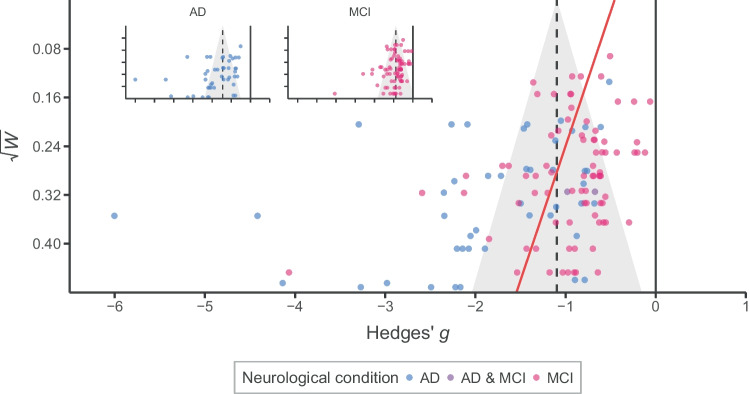
Funnel plot of the included studies. The dashed line is the overall effect size, whereas the red line represents the asymmetry in the distribution of effect sizes in terms of their corrected standard error (i.e. fitted meta-regressive coefficient of $\sqrt{W}$)

Another explanation could come from the reporting process itself, favouring the publication of positive and significant over null results (Mathur & VanderWeele, 2020). Thus, smaller studies tended to report higher effect sizes (*p* = .001; Table 3), which is evidence of publication bias in the literature. Publication bias has been progressively reduced across years ($\sqrt{W}\times$ Year of publication, *p* = .033) and among studies with MCI patients ($\sqrt{W}\times$ Neurological condition, *p* = .002; Fig. 5). Although some studies published since 2012 had large samples of patients (such as Kinsella et al., 2016; Thompson et al., 2017; Tse et al., 2015; Wang et al., 2012; *n* > 100), the sample sizes remained similar across years, especially if we removed these four exceptions, *r* = .10 [−.16, .35], *p* = .465 [mean of 31.6 patients before 2012 vs. mean of 35.8 after 2012, *t*(57.38) =  −0.461, *p* = .646; Fig. 4C, D]. Nevertheless, the sampling error decreased, *r* =  −.27 [−.48, −.02], *p* = .036 [mean *SE*_*g*_ = 0.37 before 2012 vs. mean *SE*_*g*_ = 0.33 after 2012, *t*(58.01) = 1.05, *p* = .300; Fig. 4E, F], in part because of PM tasks in the studies included more number of trials,* r* = .29 [.05, .51], *p* = .021. Whereas the studies used a mean of 4.4 trials per PM measure before the publication of the meta-analysis by van den Berg et al., the mean after that increased to 7.5 [*t*(53.95) =  −2.62, *p* = .011; Fig. 4G, H]. Therefore, selective reporting might be more likely with designs with less precision, producing less stable estimates of the effect size and with more room for outliers to appear. Finally, the use of classic neuropsychological PM tests has decreased across years, *r* =  −.27 [−.48, −.02], *p* = .036 (29% of the studies used classic neuropsychological tests before 2012 vs. 18% after 2012).

Finally, the best meta-regressive model after a backward stepwise selection with all the prespecified moderators included neurological condition, type of PM measure, and small-study effect ($\sqrt{W}$), with a residual heterogeneity of τ^2^ = 0.15 and *I*^2^ = 66.33% (vs. the heterogeneity of the model without moderators: τ^2^ = 0.22 and *I*^2^ = 76.47%). It is relevant to note that after the inclusion of these three main moderators, the variable year of publication was no longer a significant predictor, and the backward selection excluded it from the model. This supports the idea that it was the changes over the years and not the year of publication per se that explains the overall decrease in effect size.

## Discussion

Adequate remembering to take medication or turning on time to the next doctor appointment are two examples of everyday activities that are fundamental for independent living in particular for older adult individuals. These activities are also two reasonable PM tasks in everyday situations. PM failures are frequently observed in older adults (Henry et al., 2004; Kliegel et al., 2016) indeed forgetting intentions and struggling with planning actions comprised between 50 to 80% of all reported memory problems in healthy adults (Cohen et al., 2019). Considering that the number of older adults is drastically increasing over the next decades, it is timely to understand how PM decline as one gets older and identify early signs of cognitive more severe decline.

In a previous meta-analysis, van den Berg and colleagues (2012) investigated event-based and time-based PM in healthy older adults and patients with different degrees of cognitive decline. The meta-analysis included 14 studies (seven with AD patients, four with MCI, and three with both types of patients) and surprisingly showed no statistical difference between the impairment in MCI and AD. In the present work, we updated the review of the literature and meta-analysed 46 studies of PM in AD patients (10 new studies), in people with MCI (20 new), or in both groups of patients (2 new). The results of this larger sample of studies confirmed the previous finding of a lower PM performance in patients of both neurological conditions, although, this impairment was more pronounced in AD compared to MCI patients. The difference arose even when AD patients were compared with MCI patients within the same studies (Kazui et al., 2005; Thompson et al., 2010, 2011; Troyer & Murphy 2007) or when patients were contrasted against healthy older adults in studies using classic neuropsychological PM tests (i.e. CAMPROMPT, MIST, RBMT, and RPA-ProMem). These results suggest that although the deficits in PM are already observable in MCI, there is a progression in the decline throughout the advance of the disorder to AD. One clear explanation for the discrepancy between our meta-analysis and the one by van den Berg et al. is the increased amount of evidence that redounded in increased statistical power, allowing us to detect a significant difference. In addition, we have observed an increasing interest in studying PM performance in ageing and in clinical populations (Kliegel, Jager, et al., 2008; Kliegel, McDaniel, et al., 2008; Raskin, 2018) that mirrors the great interest in understanding the causes of PM impairment and monitoring the decline as an early sign of more severe neurological disorders (Hering et al., 2018). Indeed, before 2012, only seven studies have been conducted about PM in MCI patients, but since that year the number of papers has almost tripled. In the present meta-analysis, this trend has been determinant to show that MCI is characterized by the presence of PM deficits, but significantly smaller than in AD. Increasing the knowledge concerning the preclinical phase of AD is important for theoretical and clinical reasons. From a theoretical point of view, advancing the knowledge regarding the transition from normal ageing to dementia is vital in understanding how the disease evolves. From a clinical perspective, identifying individuals at risk for developing AD as early as possible is timely for boosting treatment efficacy.

Our results also indicated no conclusive evidence for an effect of the type of cue on PM performance. By definition, it is assumed that time-based PM relies more on internal, self-initiated control mechanisms than event-based PM because no external cue prompts the action (Kliegel, Jager, et al., 2008; Kliegel, McDaniel, et al., 2008). Following this definition, time-based PM performance should be particularly affected by an age-related decline (Vanneste et al., 2016). However, our results did not confirm this assumption for MCI patients, who showed similar impairment in both PM paradigms (event-based, −0.83; vs. time-based, −0.90; *BF*_10_ = 0.09), and only numerically for AD patients (event-based, −1.42; vs. time-based, −2.84; *BF*_10_ = 0.97). While the lack of conclusive evidence in the case of AD patients might be a matter of the lack of studies using time-based PM tasks in this population (only five studies), our findings suggest that a more severe cognitive deficit is necessary to cause a differential affectation of time-based PM. It is possible that previously observed differences between event-based and time-based PM tasks were merely due to differences in task characteristics rather than the difference in the type of cue for action. According to the Multiprocess framework (McDaniel & Einstein, 2000), PM performance relies on both strategic monitoring and automatic retrieval processes, based on this assumption both event-based and time-based PM tasks can vary in the amount of self-initiated processes indeed in both cases individuals are required to monitor the environment for the cue. For example, by varying the cue focality, certain event-based PM tasks may be more demanding than some time-based PM tasks, therefore the observed differences in PM performance between event-based and time-based tasks might be mainly determined by the type of process (i.e. automatic vs. controlled) rather than by the type of cue. Furthermore, although the available evidence today is substantially greater than it was a decade ago, the number of studies investigating time-based PM is still small compared to the studies investigating event-based PM.

The lack of differences in time-based and event-based PM performance was also observed in the previous meta-analysis conducted by van den Berg et al. (2012) and other meta-analyses conducted on patients with traumatic brain injury (Shum et al., 2011) and patients with Parkinson’s disease (Ramanan & Kumar, 2013). As mentioned, the age and clinical invariance for event- and time-based PM may be due to methodological differences between the measures used to detect event-based and time-based PM performance. More studies including both event-based and time-based cues are needed to better understand the specificity of these two processes and if are differently affected in healthy and pathological ageing.

One of the most striking findings of our meta-analysis was an observed reduction of the PM deficits shown by MCI and AD patients. Considering the short period that has passed since 2012, we believe that this result was not the result of a change in the effect of both neurological conditions. Instead, we detected several aspects that changed in the literature in the last decade that can account for this trend. Across years, the number of items or trials per task has increased, giving greater reliability to the PM measure and it would subsequently result in a more stable estimation of the between-group difference. In parallel, the small-study effect, a sign of potential publication bias, has been reduced in the last decade. Taking into account both correlations, we propose the use of more reliable research designs as one plausible explanation for the reduction of PM impairments. Thus, it might have produced conditions less favourable for the publication of extreme values (i.e. more stable estimates). These findings highlight the relevance of collecting enough observations per participant for getting reliable results. However, increasing the number of observations per task should not mean a drastic shortening of the intervals separating PM cues (below 2 min) at the risk of tapping short-term rather than prospective memory. Such a modification could alter the nature of the task and would prevent distinguishing whether the overall reduction of PM deficits is a consequence of higher reliability or a loss of sensitivity. The fact that most of the tasks have not exceeded that threshold, including those in the recent literature, and that the *year-of-publication effect* also appears with the studies that used classic neuropsychological tests, β = 0.05 [0.02, 0.08], *p* = .011, which have not undergone changes in their number of trials, rules out the possibility that a loss of sensitivity has been the main explanation for our finding. Reliability can also be enhanced by including multiple assessment sessions, which is more costly but feasible in institutionalized settings. Neuropsychological studies, which often experience difficulties in accessing samples, should ensure that the information they obtain from their participants is sufficient to achieve meaningful results.

Furthermore, other factors can explain the observed heterogeneity between the studies, such as the PM paradigms used. Although all the studies used laboratory-based paradigms, some of them included classical event-based or time-based tasks in which participants were engaged in an ongoing task (i.e. word categorization, Chi et al., 2014; Duchek et al., 2006) and also instructed to press a key when the designed word appeared on the screen or when a specified amount of time has passed. Other studies used a computerized task that resembles everyday activities (i.e. Virtual Week; Shelton et al., 2016; Thompson et al., 2010, 2011). The advantage of using the latter tasks concerns the possibility of using computerized controlled tasks with good psychometric properties and, at the same time, a high resemblance to real-life situations. Other studies employed PM tasks commonly used in the clinical setting. The RBMT (Huppert & Beardsall, 1993; Kazui et al., 2005; Martins & Damasceno, 2008; Mori & Sugimura, 2007) is one of the first tools used in clinical and experimental settings to investigate PM, representing a valid measure of “everyday” memory function [but Shum et al. (2002) concluded that there was little evidence to support the reliability or validity of the PM items separately]. New and more reliable tasks have been developed to be used in clinical settings such as the CAMPROMPT (Delprado et al., 2012; Dermody et al., 2016), the MIST (Belmar et al., 2020; Karantzoulis et al., 2009); the RPA-ProMem (Aronov et al, 2015; Rabin et al., 2014). All of them include both event-based and time-based activities to be performed during one session lasting 20–30 min approximately (Mioni et al., 2022). Interestingly, the observed PM impairments were larger with these neuropsychological tests compared to the rest of tasks (*g* =  −1.44 vs. −0.98), which could be partially explained by their reduced number of observations/trials [2.6 vs. 6.6, *t*(124.80) = 5.82, *p* < .001] and, on the other hand, by their potential great sensitivity to PM deficits, as they were expressly designed and validated for that purpose. Further studies that included both types of paradigms will be crucial for elucidating this result.

It is also important to consider the heterogeneity of the characteristics of patients recruited, and the methods to classify patients. Concerning the studies that include AD patients only two studies considered the different degrees of patients’ cognitive decline (Huppert & Beardsall, 1993; Tse et al., 2015). Concerning MCI patients, only five studies considered the heterogeneity of this neurological condition (e.g. amnestic or non-amnestic; single domain or multiple domains; Chi et al., 2014; Costa et al., 2015; Rabin et al., 2014; Schmitter-Edgecombe et al., 2009; Wang et al., 2012). These factors may affect the comparison between the studies and the possibility to generalize the meta-analytic result to MCI patients. It is also important to point out that in most cases that the Mini-Mental Examination State (MMSE) was the measure to evaluate global cognitive function, with few exceptions such as the Addenbrooke’s Cognitive Examination Revised (Dermody et al., 2016; Kamminga et al., 2014), the Wechsler Adult Intelligence Scale (Duchek et al., 2006), and the Montreal Cognitive Assessment (Kinsella et al., 2016; Lajeunesse et al., 2021, 2022). The MMSE is well-known and extensively used in clinical and experimental settings, but it is important to consider that it has been demonstrated to be less sensitive to detecting early manifestations of cognitive decline than the other measures used (Bergeron et al., 2017).

## Conclusions

The global population is ageing at an unprecedented rate; the number of people aged 60 and over is projected to more than double by 2050, and the number of people aged 80 and over is projected to quadruple. The ageing population is likely to have a significant impact on society, including increased demand for healthcare and long-term care services, as well as changes in the labour market and patterns of consumption. Memory complaints are the most common causes of age-related cognitive dysfunction as we age. Interest in subjective memory complaints and specifically PM complaints as possible indicators of impending dementia has increased in recent years as research focus has shifted toward identifying at the earliest possible stage people who will develop more severe forms of dementia. Consequently, the proportion of studies investigating MCI has increased in the last decade. The present work confirmed that MCI patients already showed lower PM abilities than healthy older adults, and the PM impairments increase when MCI progresses to AD. There was no difference between the deficits in time-based and event-based PM tasks for both MCI and AD patients. Although it needs further research, PM deficits were numerically larger in patients with deficits in episodic memory, such as amnestic MCI. Furthermore, the use of more reliable research designs could explain the reduction of observed PM impairments in recent years. Our findings highlight the relevance of collecting enough observations per participant for getting reliable results.

## Supplementary Information

Below is the link to the electronic supplementary material.

Supplementary file1 (DOCX 38 KB)

Supplementary file2 (DOCX 42 KB)

## Data Availability

All the data and R script for the analyses are fully available at https://osf.io/fg2zu/.

## Footnotes

1 van den Berg et al. (2012) reported on page 708 that 13 studies were included in the meta-analysis and 2 of them included data on both AD and MCI (Thompson et al., 2010; Troyer & Murphy, 2007). Looking at the tables provided on pages 709 and 711, the studies included were actually 14 and 3 of them included data on both AD and MCI (Kazui et al., 2005; Thompson et al., 2010; Troyer & Murphy, 2007). From now on when referring to the studies included in van den Berg et al. (2012), we consider the correct number of 14 studies included.

2 The MMSE score of all the samples of patients that reported it (*n* = 50) was standardized to get a moderating variable centred at 0 (across-sample mean MMSE score = 24.83 and *SD* = 2.95).

3 The standardized mean difference contrasting the score of patients with that of controls (i.e. Hedges’ *g*, defined by Eq. 1) in each of the selected cognitive domains (retrospective memory, executive functions, working memory, and processing speed) was used as a moderator.

4 The category classic neuropsychological PM tasks included the Cambridge Prospective Memory Test (CAMPROMPT, Wilson et al., 2005), the Memory for Intentions Screening Test (MIST, Raskin, 2009), the Rivermead Behavioral Memory Test (RBMT, Wilson et al., 1989), and the Royal Prince Alfred Prospective Memory Test (RPA-ProMem, Radford et al., 2011). Other PM tasks were in most cases new tasks specifically designed for the individual study or with a short history in the literature.

5 Martins and Damasceno (2012) reported the PM findings with the same sample of patients as Martins and Damasceno (2008). To avoid redundancies, we included only the first of the two studies.

6 The multilevel Bayesian approach was fitted using the *brms* R package (Bürkner, 2017) with an event-based intercept prior slightly larger for AD than for MCI patients (prior *g* =  −1.3 vs. prior *g* =  −0.8, respectively; *SD* = 1 for both models). The true heterogeneity parameter (τ) was assumed to have a half-Cauchy distribution (centred on 0 and scale γ = 2). Finally, the meta-regressive coefficient for time-based PM was assumed to follow a normal distribution with centre at 0 and a *SD* of 1.

## References

[CR1] Albert, M. S., DeKosky, S. T., Dickson, D., Dubois, B., Feldman, H. H., Fox, N. C., & Phelps, C. H. (2011). The diagnosis of mild cognitive impairment due to Alzheimer’s disease: recommendations from the National Institute on Aging‐Alzheimer’s Association workgroups on diagnostic guidelines for Alzheimer’s disease. *Alzheimer's & Dementia, 7(*3), 270–279. 10.1016/j.jalz.2011.03.008 10.1016/j.jalz.2011.03.008 PMC3312027 21514249

[CR2] Arnáiz, E., & Almkvist, O. (2003). Neuropsychological features of mild cognitive impairment and preclinical Alzheimer’s disease. *Acta Neurologica Scandinavica,**107*, 34–41. 10.1034/j.1600-0404.107.s179.7.x 12603249

[CR3] **Aronov, A., Rabin, L. A., Fogel, J., Chi, S. Y., Kann, S. J., Abdelhak, N., & Zimmerman, M. E. (2015). Relationship of cognitive strategy use to prospective memory performance in a diverse sample of nondemented older adults with varying degrees of cognitive complaints and impairment**. ***Aging, Neuropsychology, and Cognition, 22*****(4), 486–501**. 10.1080/13825585.2014.984653 10.1080/13825585.2014.984653 PMC4624306 25471537

[CR4] Bäckman, L., Jones, S., Berger, A. K., Laukka, E. J., & Small, B. J. (2005). Cognitive impairment in preclinical Alzheimer’s disease: A meta-analysis. *Neuropsychology,**19*(4), 520–531. 10.1037/0894-4105.19.4.520 16060827 10.1037/0894-4105.19.4.520

[CR5] Baddeley, A. D., Baddeley, H. A., Bucks, R. S., & Wilcock, G. K. (2001). Attentional control in Alzheimer’s disease. *Brain,**124*(8), 1492–1508. 10.1093/brain/124.8.1492 11459742 10.1093/brain/124.8.1492

[CR6] Bastin, C., Delhaye, E., Moulin, C., & Barbeau, E. J. (2019). Novelty processing and memory impairment in Alzheimer’s disease: A review. *Neuroscience & Biobehavioral Reviews,**100*, 237–249. 10.1016/j.neubiorev.2019.02.021 30851282 10.1016/j.neubiorev.2019.02.021

[CR7] **Beaver, J., & Schmitter-Edgecombe, M. (2017). Multiple types of memory and everyday functional assessment in older adults.*****Archives of Clinical Neuropsychology,******32*****(4), 413–426.**10.1093/arclin/acx016 28334170 10.1093/arclin/acx016 PMC5439212

[CR8] **Belmar, M., Gladwin, T. E., Reis, L., Pinho, M. S., Silva, D., Nunes, M. V., & Pereira, A. (2020). An exploration of prospective memory components and subtasks of the Memory for Intentions Test (MIST).** ***Journal of Clinical and Experimental Neuropsychology***, ***42*****(3), 274–284**. 10.1080/13803395.2019.1710111 10.1080/13803395.2019.1710111 31937187

[CR9] Bergeron, D., Flynn, K., Verret, L., Poulin, S., Bouchard, R. W., Bocti, C., & Laforce, R. J. (2017). Multicenter validation of an MMSE-MoCA conversion table. *Journal of the American Geriatrics Society, 65*(5), 1067–1072. 10.1111/jgs.14779 10.1111/jgs.14779 28205215

[CR10] **Blanco-Campal, A., Coen, R. F., Lawlor, B. A., Walsh, J. B., & Burke, T. E. (2009). Detection of prospective memory deficits in mild cognitive impairment of suspected Alzheimer’s disease etiology using a novel event-based prospective memory task.*****Journal of the International Neuropsychological Society,******15*****(1), 154–159.**10.1017/S1355617708090127 19128540 10.1017/S1355617708090127

[CR11] Borenstein, M., Hedges, L. V., Higgins, J. P., & Rothstein, H. R. (2021). *Introduction to meta-analysis*. John Wiley & Sons.

[CR12] Burgess, P. W., Dumontheil, I., & Gilbert, S. J. (2007). The gateway hypothesis of rostral prefrontal cortex (area 10) function. *Trends in Cognitive Sciences,**11*, 290–298. 10.1016/j.tics.2007.05.004 17548231 10.1016/j.tics.2007.05.004

[CR13] Burgess, P. W., Quayle, A., & Frith, C. D. (2001). Brain regions involved in prospective memory as determined by positron emission tomography. *Neuropsychologia,**39*, 545–555. 10.1016/S0028-3932(00)00149-4 11257280 10.1016/s0028-3932(00)00149-4

[CR14] Burgess, P. W., Scott, S. K., & Frith, C. D. (2003). The role of the rostral frontal cortex (area 10) in prospective memory: A lateral versus medial dissociation. *Neuropsychologia,**41*, 906–918. 10.1016/S0028-3932(02)00327-5 12667527 10.1016/s0028-3932(02)00327-5

[CR15] Burgess, P. W., & Shallice, T. (1997). The relationship between prospective and retrospective memory: Neuropsychological evidence. In M. A. Conway (Ed.), *Cognitive Models of Memory,* (pp. 247–272). The MIT Press.

[CR16] Bürkner, P. (2017). brms: An R package for Bayesian multilevel models using stan. *Journal of Statistical Software, 80*(1), 1–28. 10.18637/jss.v080.i01

[CR17] **Cheng, L., Tu, M. C., Huang, W. H., & Hsu, Y. H. (2021). Effects of mental imagery on prospective memory: A process analysis in individuals with amnestic mild cognitive impairment.*****Gerontology,******67*****(5), 644–654.**10.1159/000514869 10.1159/000514869 33853071

[CR18] **Chi, S. Y., Rabin, L. A., Aronov, A., Fogel, J., Kapoor, A., & Wang, C. (2014). Differential focal and nonfocal prospective memory accuracy in a demographically diverse group of nondemented community-dwelling older adults.*****Journal of the International Neuropsychological Society,******20*****(10), 1015–1027.**10.1017/S1355617714000964 25401793 10.1017/S1355617714000964 PMC4624305

[CR19] Cohen, R. A., Marsiske, M. M., & Smith, G. E. (2019). Neuropsychology of aging. *Handbook of Clinical Neurology,**167*, 149–180. 10.1016/B978-0-12-804766-8.00010-8 31753131 10.1016/B978-0-12-804766-8.00010-8

[CR20] Cona, G., Scarpazza, C., Sartori, G., Moscovitch, M., & Bisiacchi, P. S. (2015). Neural bases of prospective memory: A meta-analysis and the “Attention to Delayed Intention” (AtoDI) model. *Neuroscience & Biobehavioral Reviews,**52*, 21–37. 25704073 10.1016/j.neubiorev.2015.02.007

[CR21] **Costa, A., Perri, R., Serra, L., Barban, F., Gatto, I., Zabberoni, S., & Carlesimo, G. A. (2010). Prospective memory functioning in mild cognitive impairment.*****Neuropsychology, 24*****(3), 327–335.**10.1037/a0018015 10.1037/a0018015 20438210

[CR22] **Costa, A., Fadda, L., Perri, R., Brisindi, M., Lombardi, M. G., Caltagirone, C., & Carlesimo, G. A. (2015). Sensitivity of a time-based prospective memory procedure in the assessment of amnestic mild cognitive impairment.*****Journal of Alzheimer’s Disease,******44*****(1), 63–67.**10.3233/JAD-142070 25208618 10.3233/JAD-142070

[CR23] **Costa, A., Perri, R., Zabberoni, S., Barban, F., Caltagirone, C., & Carlesimo, G. A. (2011). Event-based prospective memory failure in amnestic mild cognitive impairment.*****Neuropsychologia,******49*****(8), 2209–2216.**10.1016/j.neuropsychologia.2011.03.016 21419790 10.1016/j.neuropsychologia.2011.03.016

[CR24] **Crook-Rumsey, M., Howard, C. J., Hadjiefthyvoulou, F., & Sumich, A. (2022). Neurophysiological markers of prospective memory and working memory in typical ageing and mild cognitive impairment.*****Clinical Neurophysiology,******133***, **111–125.**10.1016/j.clinph.2021.09.019 34839236 10.1016/j.clinph.2021.09.019

[CR25] de Vrij, F. M., Fischer, D. F., van Leeuwen, F. W., & Hol, E. M. (2004). Protein quality control in Alzheimer’s disease by the ubiquitin proteasome system. *Progress in Neurobiology,**74*(5), 249–270. 15582222 10.1016/j.pneurobio.2004.10.001

[CR26] **Delprado, J., Kinsella, G., Ong, B., Pike, K., Ames, D., Storey, E., ... & Rand, E. (2012). Clinical measures of prospective memory in amnestic mild cognitive impairment.** ***Journal of the International Neuropsychological Society***, ***18*****(2), 295–304.**10.1017/S135561771100172X 10.1017/S135561771100172X 22264396

[CR27] **Dermody, N., Hornberger, M., Piguet, O., Hodges, J. R., & Irish, M. (2016). Prospective memory impairments in Alzheimer’s disease and behavioral variant frontotemporal dementia: Clinical and neural correlates.*****Journal of Alzheimer’s Disease,******50*****(2), 425–441.**10.3233/JAD-150871 26682697 10.3233/JAD-150871

[CR28] Díaz-Mardomingo, M. D. C., García-Herranz, S., Rodríguez-Fernández, R., Venero, C., & Peraita, H. (2017). Problems in classifying mild cognitive impairment (MCI): One or multiple syndromes? *Brain Sciences,**7*(9), 111. 10.3390/brainsci7090111 28862676 10.3390/brainsci7090111 PMC5615252

[CR29] **Duchek, J. M., Balota, D. A., & Cortese, M. (2006). Prospective memory and apolipoprotein E in healthy aging and early stage Alzheimer’s disease.*****Neuropsychology,******20*****(6), 633–644.**10.1037/0894-4105.20.6.633 17100508 10.1037/0894-4105.20.6.633

[CR30] Egger, M., Smith, D. G., Schneider, M., & Minder, C. (1997). Bias in meta-analysis detected by a simple, graphical test. *BMJ,**315*, 629–634. 10.1136/bmj.315.7109.629 9310563 10.1136/bmj.315.7109.629 PMC2127453

[CR31] **El Haj, M., Coello, Y., Kapogiannis, D., Gallouj, K., & Antoine, P. (2018). Negative prospective memory in Alzheimer’s disease: “Do not perform that action.”*****Journal of Alzheimer’s Disease,******61*****(2), 663–672.**10.3233/JAD-170807 29226877 10.3233/JAD-170807 PMC5925753

[CR32] Ellis, J., & Kvavilashvili, L. (2000). Prospective memory in 2000: Past, present, and future directions. *Applied Cognitive Psychology,**14*, 1–9. 10.1002/acp.767

[CR33] **Farina, N., Young, J., Tabet, N., & Rusted, J. (2013). Prospective memory in Alzheimer-type dementia: Exploring prospective memory performance in an age-stratified sample.*****Journal of Clinical and Experimental Neuropsychology,******35*****(9), 983–992.**10.1080/13803395.2013.844772 24131030 10.1080/13803395.2013.844772

[CR34] Fisher, Z., Tipton, E., & Zhipeng, H. (2017). robumeta: Robust variance meta-regression. R package version 2.0. https://cran.r-project.org/web/packages/robumeta/

[CR35] **Gao, J. L., Cheung, R. T. F., Chan, Y. S., Chu, L. W., & Lee, T. M. C. (2013). Increased prospective memory interference in normal and pathological aging: Different roles of motor and verbal processing speed.*****Aging, Neuropsychology, and Cognition,******20*****(1), 80–100.**10.1080/13825585.2012.672948 10.1080/13825585.2012.672948 22486785

[CR36] Glisky, E. L. (2007). Changes in cognitive function in human aging. In D. R. Riddle (Ed.), Brain aging: Models, methods, and mechanisms (pp. 3–20). CRC Press/Routledge/Taylor & Francis Group. 10.1201/9781420005523.sec1

[CR37] Gonneaud, J., Rauchs, G., Groussard, M., Landeau, B., Mézenge, F., de La Sayette, V., et al. (2014). How do we process event-based and time-based intentions in the brain? An fMRI study of prospective memory in healthy individuals. *Human Brain Mapping,**35*, 3066–3082. 10.1002/hbm.22385 24214215 10.1002/hbm.22385 PMC6869434

[CR38] Hedges, L. V., Tipton, E., & Johnson, M. C. (2010). Robust variance estimation in meta-regression with dependent effect size estimates. *Research Synthesis Methods,**1*(1), 39–65. 26056092 10.1002/jrsm.5

[CR39] Henry, J. D., MacLeod, M. S., Phillips, L. H., & Crawford, J. R. (2004). A meta-analytic review of prospective memory and aging. *Psychology and Aging,**19*(1), 27–39. 10.1037/0882-7974.19.1.27 15065929 10.1037/0882-7974.19.1.27

[CR40] Hering, A., Kliegel, M., Rendell, P. G., Craik, F. I., & Rose, N. S. (2018). Prospective memory is a key predictor of functional independence in older adults. *Journal of the International Neuropsychological Society,**24*(6), 640–645. 10.1017/S1355617718000152 29606153 10.1017/S1355617718000152

[CR41] **Huppert, F. A., & Beardsall, L. (1993). Prospective memory impairment as an early indicator of dementia.*****Journal of Clinical and Experimental Neuropsychology,******15*****(5), 805–821.**10.1080/01688639308402597 8276937 10.1080/01688639308402597

[CR42] **Jones, S., Livner, Å., & Bäckman, L. (2006). Patterns of prospective and retrospective memory impairment in preclinical Alzheimer’s disease.*****Neuropsychology,******20*****(2), 144–152.**10.1037/0894-4105.20.2.144 16594775 10.1037/0894-4105.20.2.144

[CR43] **Kamminga, J., O’Callaghan, C., Hodges, J. R., & Irish, M. (2014). Differential prospective memory profiles in frontotemporal dementia syndromes.*****Journal of Alzheimer’s Disease,******38*****(3), 669–679.**10.3233/JAD-131118 24056089 10.3233/JAD-131118

[CR44] **Karantzoulis, S., Troyer, A. K., & Rich, J. B. (2009). Prospective memory in amnestic mild cognitive impairment.*****Journal of the International Neuropsychological Society,******15*****(3), 407–415.**10.1017/S1355617709090596 19402927 10.1017/S1355617709090596

[CR45] **Kazui, H., Matsuda, A., Hirono, N., Mori, E., Miyoshi, N., Ogino, A., & Takeda, M. (2005). Everyday memory impairment of patients with mild cognitive impairment.*****Dementia and Geriatric Cognitive Disorders, 19*****(5–6), 331–337.**10.1159/000084559 10.1159/000084559 15785034

[CR46] **Kinsella, G. J., Ames, D., Storey, E., Ong, B., Pike, K. E., Saling, M. M., & Rand, E. (2016). Strategies for improving memory: A randomized trial of memory groups for older people, including those with mild cognitive impairment.** ***Journal of Alzheimer's Disease***, ***49*****(1), 31–43.**10.3233/JAD-150378 10.3233/JAD-150378 26444773

[CR47] Kinsella, G. J., Ong, B., Storey, E., Wallace, J., & Hester, R. (2007). Elaborated spaced-retrieval and prospective memory in mild Alzheimer’s disease. *Neuropsychological Rehabilitation,**17*(6), 688–706. 10.1080/09602010600892824 17852763 10.1080/09602010600892824

[CR48] Kliegel, M., McDaniel, M. A., & Einstein, G. O. (Eds.). (2008). *Prospective Memory: Cognitive, Neuroscience, Developmental, and Applied Perspectives*. Psychology Press.

[CR49] Kliegel, M., Ballhausen, N., Hering, A., Ihle, A., Schnitzspahn, K. M., & Zuber, S. (2016). Prospective memory in older adults: Where we are now and what is next. *Gerontology,**62*(4), 459–466. 10.1159/000443698 26950339 10.1159/000443698

[CR50] Kliegel, M., Jäger, T., Altgassen, M., & Shum, D. (2008). Clinical neuropsychology of prospective memory. In M. Kliegel, M. A. McDaniel, & G. O. Einstein (Eds.), *Prospective memory: Cognitive, Neuroscience, Developmental, and Applied Perspectives* (pp. 283–308). Taylor & Francis Group/Lawrence Erlbaum Associates.

[CR51] Laera, G., Joly-Burra, E., Zuber, S., Ballhausen, N., Künzi, M., Ihle, A., & Hering, A. (2021). Do executive functions explain older adults’ health-related quality of life beyond event-based prospective memory? *Aging, Neuropsychology, and Cognition,* 1–15. 10.1080/13825585.2021.1989368 10.1080/13825585.2021.1989368 34665685

[CR52] **Lajeunesse, A., Potvin, M. J., Labelle, V., Chasles, M. J., Kergoat, M. J., Villalpando, J. M., ... & Rouleau, I. (2022). Effectiveness of a visual imagery training program to improve prospective memory in older adults with and without mild cognitive impairment: A randomized controlled study.** ***Neuropsychological Rehabilitation***, ***32*****(7), 1576–1604.**10.1080/09602011.2021.1919529 10.1080/09602011.2021.1919529 33947319

[CR53] **Lajeunesse, A., Potvin, M. J., Labelle, V., Joubert, S., & Rouleau, I. (2021). Characterization of prospective memory in mild cognitive impairment by using the Ecological test of Prospective Memory.*****Aging, Neuropsychology, and Cognition,******28*****(3), 367–391.**10.1080/13825585.2020.1772192 10.1080/13825585.2020.1772192 32487003

[CR54] Lamichhane, B., McDaniel, M. A., Waldum, E. R., & Braver, T. S. (2018). Age-related changes in neural mechanisms of prospective memory. *Cognitive Affective Behavioural Neuroscience,**18*, 982–999. 10.3758/s13415-018-0617-1 10.3758/s13415-018-0617-1 PMC6309350 29926283

[CR55] **Lecouvey, G., Morand, A., Gonneaud, J., Piolino, P., Orriols, E., Pélerin, A., & Desgranges, B. (2019). An impairment of prospective memory in mild Alzheimer’s disease: A ride in a virtual town.** ***Frontiers in Psychology***, ***10***, **241.**10.3389/fpsyg.2019.00241 10.3389/fpsyg.2019.00241 PMC6379453 30809174

[CR56] **Lee, J. H., Shelton, J. T., Scullin, M. K., & McDaniel, M. A. (2016). An implementation intention strategy can improve prospective memory in older adults with very mild Alzheimer’s disease.*****British Journal of Clinical Psychology,******55*****(2), 154–166.**10.1111/bjc.12084 25994043 10.1111/bjc.12084 PMC4654698

[CR57] Martin, M., Kliegel, M., & McDaniel, M. A. (2003). The involvement of executive functions in prospective memory performance of adults. *International Journal of Psychology,**38*(4), 195–206. 10.1080/00207590344000123

[CR58] **Martins, S. P., & Damasceno, B. P. (2008). Prospective and retrospective memory in mild Alzheimer’s disease.*****Arquivos De Neuro-Psiquiatria,******66***, **318–322.**10.1590/S0004-282X2008000300006 18641863 10.1590/s0004-282x2008000300006

[CR59] **Martins, S. P., & Damasceno, B. P. (2012). Accuracy of prospective memory tests in mild Alzheimer’s disease.*****Arquivos De Neuro-Psiquiatria,******70***, **17–21.**10.1590/S0004-282X2012000100005 22218468 10.1590/s0004-282x2012000100005

[CR60] **Massa, F., Grisanti, S., Brugnolo, A., Doglione, E., Orso, B., Morbelli, S., & Girtler, N. (2020). The role of anterior prefrontal cortex in prospective memory: An exploratory FDG-PET study in early Alzheimer’s disease.** ***Neurobiology of Aging***, ***96***, **117–127.**10.1016/j.neurobiolaging.2020.09.003 10.1016/j.neurobiolaging.2020.09.003 33002765

[CR61] Mathur, M. B., & VanderWeele, T. J. (2020). Sensitivity analysis for publication bias in meta-analyses. *Journal of the Royal Statistical Society: Series C (applied Statistics),**69*(5), 1091–1119. 10.1111/rssc.12440 33132447 10.1111/rssc.12440 PMC7590147

[CR62] **Maylor, E. A., Smith, G., Sala, S. D., & Logie, R. H. (2002). Prospective and retrospective memory in normal aging and dementia: An experimental study.*****Memory & Cognition,******30*****(6), 871–884.**10.3758/BF03195773 12450091 10.3758/bf03195773

[CR63] McDaniel, M. A., & Einstein, G. O. (2007). Prospective memory: An overview and synthesis of an emerging field. Sage Publications, Inc.

[CR64] McDaniel, M. A., & Einstein, G. O. (2000). Strategic and automatic processes in prospective memory retrieval: A multiprocess framework. *Applied Cognitive Psychology*. *Special Issue: New Perspectives in Prospective Memory,**14*, S127–S144. 10.1002/acp.775

[CR65] McDaniel, M. A., & Einstein, G. O. (2011). The neuropsychology of prospective memory in normal aging: A componential approach. *Neuropsychologia,**49*(8), 2147–2155. 10.1016/j.neuropsychologia.2010.12.029 21192957 10.1016/j.neuropsychologia.2010.12.029 PMC3095717

[CR66] McFarland, C. P., & Glisky, E. L. (2009). Frontal lobe involvement in a task of time-based prospective memory. *Neuropsychologia,**47*(7), 1660–1669. 10.1016/j.neuropsychologia.2009.02.023 19397861 10.1016/j.neuropsychologia.2009.02.023 PMC2691905

[CR67] McKhann, G. M., Knopman, D. S., Chertkow, H., Hyman, B. T., Jack Jr, C. R., Kawas, C. H., & Phelps, C. H. (2011). The diagnosis of dementia due to Alzheimer’s disease: Recommendations from the National Institute on Aging-Alzheimer’s Association workgroups on diagnostic guidelines for Alzheimer's disease. *Alzheimer's & dementia, 7*(3), 263–269. 10.1016/j.jalz.2011.03.005 PMC3312024 21514250

[CR68] Mimura, M., & Yano, M. (2006). Memory impairment and awareness of memory deficits in early-stage Alzheimer’s disease. *Reviews in the Neurosciences,**17*(1–2), 253–266. 10.1620/tjem.215.133 16703956 10.1515/revneuro.2006.17.1-2.253

[CR69] Mioni, G., Fracasso, V., Cardullo, S., & Stablum, F. (2022). Comparing different tests to detect early manifestation of prospective memory decline in aging. *The Clinical Neuropsychologist,**36*(1), 105–137. 10.1080/13854046.2020.1749308 32301378 10.1080/13854046.2020.1749308

[CR70] Moher, D., Liberati, A., Tetzlaff, J., Altman, D. G., & Prisma Group. (2009). Reprint—preferred reporting items for systematic reviews and meta-analyses: The PRISMA statement. *Physical Therapy,**89*(9), 873–880. 10.7326/0003-4819-151-4-200908180-00135 19723669

[CR71] Morand, A., Segobin, S., Lecouvey, G., Gonneaud, J., Eustache, F., Rauchs, G., & Desgranges, B. (2021). Brain substrates of time-based prospective memory decline in aging: A voxel-based morphometry and diffusion tensor imaging study. *Cerebral Cortex,**31*(1), 396–409. 32935836 10.1093/cercor/bhaa232

[CR72] **Mori, A., & Sugimura, K. (2007). Characteristics of assessment of motor and process skills and Rivermead Behavioral Memory Test in elderly women with dementia and community-dwelling women.*****Nagoya Journal of Medical Science,******69*****(1/2), 45.** 17378180

[CR73] Morishima-Kawashima, M., & Ihara, Y. (2002). Alzheimer’s disease: β-Amyloid protein and tau. *Journal of Neuroscience Research,**70*(3), 392–401. 10.1002/jnr.10355 12391602 10.1002/jnr.10355

[CR74] Murman, D. L. (2015). The impact of age on cognition. *Seminars in Hearing,**36*(3), 111–121. 10.1055/s-0035-1555115 27516712 10.1055/s-0035-1555115 PMC4906299

[CR75] **Niedźwieńska, A., Kvavilashvili, L., Ashaye, K., & Neckar, J. (2017). Spontaneous retrieval deficits in amnestic mild cognitive impairment: A case of focal event-based prospective memory.*****Neuropsychology,******31*****(7), 735–749.**10.1037/neu0000378 28406664 10.1037/neu0000378

[CR76] Okuda, J., Fujii, T., Ohtake, H., Tsukiura, T., Yamadori, A., Frith, C. D., et al. (2007). Differential involvement of regions of rostral prefrontal cortex (Brodmann area 10) in time- and event-based prospective memory. *International Journal Psychophysiology,**64*, 233–246. 10.1016/j.ijpsycho.2006.09.009 10.1016/j.ijpsycho.2006.09.009 17126435

[CR77] Page, M. J., McKenzie, J. E., Bossuyt, P. M., Boutron, I., Hoffmann, T. C., Mulrow, C. D., & Moher, D. (2021). Updating guidance for reporting systematic reviews: Development of the PRISMA 2020 statement. *Journal of Clinical Epidemiology, 134*, 103–112. 10.1016/j.jclinepi.2021.02.003 10.1016/j.jclinepi.2021.02.003 33577987

[CR78] **Pereira, A., Altgassen, M., Atchison, L., de Mendonça, A., & Ellis, J. (2018). Sustaining prospective memory functioning in amnestic mild cognitive impairment: A lifespan approach to the critical role of encoding.*****Neuropsychology,******32*****(5), 634–644.**10.1037/neu0000441 29658724 10.1037/neu0000441

[CR79] **Pereira, A., de Mendonca, A., Silva, D., Guerreiro, M., Freeman, J., & Ellis, J. (2015). Enhancing prospective memory in mild cognitive impairment: The role of enactment.*****Journal of Clinical and Experimental Neuropsychology,******37*****(8), 863–877.**10.1080/13803395.2015.1072499 26313515 10.1080/13803395.2015.1072499

[CR80] Petersen, P. E. (2003). The World Oral Health Report 2003: Continuous improvement of oral health in the 21^st^ century–The approach of the WHO Global Oral Health Programme. *Community Dentistry and Oral Epidemiology,**31*, 3–24. 10.1046/j.2003.com122.x 15015736 10.1046/j..2003.com122.x

[CR81] Petersen, R. C. (2004). Mild cognitive impairment as a diagnostic entity. *Journal of Internal Medicine,**256*(3), 183–194. 10.1111/j.1365-2796.2004.01388.x 15324362 10.1111/j.1365-2796.2004.01388.x

[CR82] Petersen, R. C., Smith, G. E., Waring, S. C., Ivnik, R. J., Tangalos, E. G., & Kokmen, E. (1999). Mild cognitive impairment: Clinical characterization and outcome. *Archives of Neurology,**56*(3), 303–308. 10.1001/archneur.56.3.303 10190820 10.1001/archneur.56.3.303

[CR83] Pustejovsky, J. E., & Rodgers, M. A. (2019). Testing for funnel plot asymmetry of standardized mean differences. *Research Synthesis Methods,**10*(1), 57–71. 10.1002/jrsm.1332 30506832 10.1002/jrsm.1332

[CR84] **Rabin, L. A., Chi, S. Y., Wang, C., Fogel, J., Kann, S. J., & Aronov, A. (2014). Prospective memory on a novel clinical task in older adults with mild cognitive impairment and subjective cognitive decline.*****Neuropsychological Rehabilitation,******24*****(6), 868–893.**10.1080/09602011.2014.915855 24875614 10.1080/09602011.2014.915855 PMC4225771

[CR85] Radford, K. A., Lah, S., Say, M. J., & Miller, L. A. (2011). Validation of a new measure of prospective memory: The Royal Prince Alfred Prospective Memory Test. *The Clinical Neuropsychologist,**25*, 127–140. 10.1080/13854046.2010.529463 21108144 10.1080/13854046.2010.529463

[CR86] Ramanan, S., & Kumar, D. (2013). Prospective memory in Parkinson’s disease: A meta-analysis. *Journal of the International Neuropsychological Society,**19*(10), 1109–1118. 24044729 10.1017/S1355617713001045

[CR87] Raskin, S. A. (2009). Memory for intentions screening test: Psychometric properties and clinical evidence. *Brain Impairment,**10*(1), 23–33. 10.1375/brim.10.1.23

[CR88] Raskin, S. A. (2018). Prospective memory in clinical populations. *The Clinical Neuropsychologist,**32*(5), 741–747. 10.1080/13854046.2018.1484519 29936902 10.1080/13854046.2018.1484519

[CR89] **Schmitter-Edgecombe, M., Woo, E., & Greeley, D. R. (2009). Characterizing multiple memory deficits and their relation to everyday functioning in individuals with mild cognitive impairment.*****Neuropsychology,******23*****(2), 168–177.**10.1037/a0014186 19254090 10.1037/a0014186

[CR90] Schnitzspahn, K. M., Stahl, C., Zeintl, M., Kaller, C. P., & Kliegel, M. (2013). The role of shifting, updating, and inhibition in prospective memory performance in young and older adults. *Developmental Psychology,**49*(8), 1544–1553. 10.1037/a0030579 23148933 10.1037/a0030579

[CR91] **Shelton, J. T., Lee, J. H., Scullin, M. K., Rose, N. S., Rendell, P. G., & McDaniel, M. A. (2016). Improving prospective memory in healthy older adults and individuals with very mild Alzheimer’s disease.*****Journal of the American Geriatrics Society,******64*****(6), 1307–1312.**10.1111/jgs.14134 27321610 10.1111/jgs.14134 PMC4976780

[CR92] Shum, D., Fleming, J., & Neulinger, K. (2002). Prospective memory and traumatic brain injury: A review. *Brain Impairment,**3*(1), 1–16. 10.1375/brim.3.1.1

[CR93] Shum, D., Levin, H., & Chan, R. C. (2011). Prospective memory in patients with closed head injury: A review. *Neuropsychologia,**49*(8), 2156–2165. 21315750 10.1016/j.neuropsychologia.2011.02.006

[CR94] Stanley, T. D., & Doucouliagos, C. H. (2014). Meta-regression approximations to reduce publication selection bias. *Research Synthesis Methods,**5*, 60–78. 10.1002/jrsm.1095 26054026 10.1002/jrsm.1095

[CR95] **Tam, J. W., & Schmitter-Edgecombe, M. (2013). Event-based prospective memory and everyday forgetting in healthy older adults and individuals with mild cognitive impairment.*****Journal of Clinical and Experimental Neuropsychology,******35*****(3), 279–290.**10.1080/13803395.2013.770823 23419059 10.1080/13803395.2013.770823 PMC3600384

[CR96] **Thompson, C., Henry, J. D., Rendell, P. G., Withall, A., & Brodaty, H. (2010). Prospective memory function in mild cognitive impairment and early dementia.*****Journal of the International Neuropsychological Society,******16*****(2), 318–325.**10.1017/S1355617709991354 20128933 10.1017/S1355617709991354

[CR97] **Thompson, C. L., Henry, J. D., Rendell, P. G., Withall, A., Kochan, N. A., Sachdev, P., & Brodaty, H. (2017). Prospective memory function and cue salience in mild cognitive impairment: Findings from the Sydney Memory and Ageing Study.*****Journal of Clinical and Experimental Neuropsychology,******39*****(10), 941–953.**10.1080/13803395.2017.1281382 28145153 10.1080/13803395.2017.1281382

[CR98] **Thompson, C. L., Henry, J. D., Withall, A., Rendell, P. G., & Brodaty, H. (2011). A naturalistic study of prospective memory function in MCI and dementia.*****British Journal of Clinical Psychology,******50*****(4), 425–434.**10.1111/j.2044-8260.2010.02004.x 22003951 10.1111/j.2044-8260.2010.02004.x

[CR99] Tipton, E. (2015). Small sample adjustments for robust variance estimation with meta-regression. *Psychological Methods,**20*(3), 375–393. 10.1037/met0000011 24773356 10.1037/met0000011

[CR100] **Troyer, A. K., & Murphy, K. J. (2007). Memory for intentions in amnestic mild cognitive impairment: Time-and event-based prospective memory.*****Journal of the International Neuropsychological Society,******13*****(2), 365–369.**10.1017/S1355617707070452 17286894 10.1017/S1355617707070452

[CR101] **Tse, C. S., Chang, J. F., Fung, A. W., Lam, L. C., Hau, K. T., Leung, G. T., & Balota, D. A. (2015). The utility of a non-verbal prospective memory measure as a sensitive marker for early-stage Alzheimer’s disease in Hong Kong.*****International Psychogeriatrics,******27*****(2), 231–242.**10.1017/S1041610214002038 25245181 10.1017/S1041610214002038

[CR102] van den Berg, E., Kant, N., & Postma, A. (2012). Remember to buy milk on the way home! A meta-analytic review of prospective memory in mild cognitive impairment and dementia. *Journal of the International Neuropsychological Society,**18*(4), 706–716. 10.1017/S1355617712000331 22595831 10.1017/S1355617712000331

[CR103] Vanneste, S., Baudouin, A., Bouazzaoui, B., & Taconnat, L. (2016). Age-related differences in time-based prospective memory: The role of time estimation in the clock monitoring strategy. *Memory,**24*(6), 812–825. 10.1080/09658211.2015.1054837 26247302 10.1080/09658211.2015.1054837

[CR104] **Wang, B., Guo, Q., Zhao, Q., & Hong, Z. (2012). Memory deficits for non-amnestic mild cognitive impairment.*****Journal of Neuropsychology,******6*****(2), 232–241.**10.1111/j.1748-6653.2011.02024.x 22305009 10.1111/j.1748-6653.2011.02024.x

[CR105] Wilson, B., Cockburn, J., Baddeley, A., & Hiorns, R. (1989). The development and validation of a test battery for detecting and monitoring everyday memory problems. *Journal of Clinical and Experimental Neuropsychology,**11*, 855–870. 10.1080/01688638908400940 2592527 10.1080/01688638908400940

[CR106] Wilson, B. A., Shiel, A., Foley, J., Emslie, H., Groot, Y., Hawkins, K. A., & y Evans, J. J. (2005). *The Cambridge Prospective Memory Test*. Harcourt.

[CR107] **Zhou, T., Broster, L. S., Jiang, Y., Bao, F., Wang, H., & Li, J. (2012). Deficits in retrospective and prospective components underlying prospective memory tasks in amnestic mild cognitive impairment.*****Behavioral and Brain Functions,******8*****(1), 1–9.**10.1186/1744-9081-8-39 22888762 10.1186/1744-9081-8-39 PMC3621687

[CR108] **Zhuang, X. M., Kuo, L. W., Lin, S. Y., Yang, J. J., Tu, M. C., & Hsu, Y. H. (2021). Prospective memory and regional functional connectivity in subcortical ischemic vascular disease.** ***Frontiers in Aging Neuroscience*****, 527.**10.3389/fnagi.2021.686040 10.3389/fnagi.2021.686040 PMC8417716 34489671

