# Supplementary material for: Time-Based and Event-Based Prospective Memory in Mild Cognitive Impairment and Alzheimer’s Disease Patients: A Systematic Review and Meta-analysis
Source: Neuropsychol Rev. 2023 Nov 14;35(1):102–25. doi: 10.1007/s11065-023-09626-y (PMC11965160; doi:10.1007/s11065-023-09626-y)
Supplement: Supplementary file 1 — Supplementary file1 (DOCX 38 KB) [file 11065_2023_9626_MOESM1_ESM.docx]

**Supplementary 1:** Tasks included in the neuropsychological evaluation for AD and controls.

|  | **Cognitive function** (task used) | **AD Patients**  **M (SD)** |  | **Controls**  **M (SD)** |
| --- | --- | --- | --- | --- |
| **Dermody et al., 2016** | **General cognitive abilities** |  |  |  |
|  | ACE-R | 67.5 (13.9) |  | 94.7 (2.8) |
|  | **Verbal episodic encoding and retrieval** |  |  |  |
|  | RAVLT delayed recall | 3.1 (3.4) |  | 10.5 (3.2) |
|  | **Verbal fluency** |  |  |  |
|  | Letter fluency (FAS) | 29.5 (18.9) |  | 47.9 (12.2) |
|  | **Non-verbal episodic delayed recall** |  |  |  |
|  | RCFT 3min recall | 4.5 (6.3) |  | 18.0 (6.1) |
|  | Doors A | 8.0 (2.2) |  | 10.9 (1.5) |
|  | **Set-switching and divided attention;** |  |  |  |
|  | TMT A (sec) | 87.7 (79.9) |  | 35.6 (11.0) |
|  | TMT B-A | 212.0 (161.5) |  | 45.1 (10.7) |
|  | **Attention/short-term working memory** |  |  |  |
|  | Digit span backwatds | 4.1 (1.4) |  | 8.1 (2.4) |
|  | **Verbal semantic performance** |  |  |  |
|  | Naming (SydBat) | 20.0 (5.0) |  | 26.4 (2.3) |
|  | Comprehension (SydBat) | 24.5 (3.4) |  | 28.7 (1.9) |
|  | **Response inhibition** |  |  |  |
|  | Hayling (tot) | 3.2 (1.7) |  | 5.9 (1.1) |
| **Duchek et al. (2006)** | **Memory** |  |  |  |
|  | WMS Logical Memory | 5.37 (2.90) |  | 11.28 (3.50) |
|  | Forward Digit Span | 6.10 (1.07) |  | 6.87 (1.18) |
|  | Backward Digit Span | 4.32 (1.01) |  | 5.55 (1.36) |
|  | Associate Memory | 9.36 (2.98) |  | 16.91 (3.32) |
|  | Associate Recognition | 6.14 (1.23) |  | 6.98 (0.08) |
|  | Mental Control | 6.12 (2.26) |  | 7.94 (1.25) |
|  | **Word Fluency Test S-P** | 23.58 (6.58) |  | 34.51 (13.61) |
|  | WAIS Information | 17.53 (5.51) |  | 22.32 (4.70) |
|  | **General intelligence** |  |  |  |
|  | Block Design | 26.20 (12.50) |  | 32.20 (6.51) |
|  | Digit Symbol | 34.53 (11.89) |  | 51.10 (12.04) |
|  | **Visual perceptual–motor performance** |  |  |  |
|  | Benton Copy Test no. correct | 9.65 (0.54) |  | 9.87 (0.29) |
|  | TMT A (sec) | 58.07 (39.20) |  | 33.96 (11.38) |
|  | TMT B (sec) | 137.87 (44.20) |  | 84.10 (31.87) |
|  | **Semantic/lexical retrieval** |  |  |  |
|  | Boston Naming Test | 48.54 (8.13) |  | 56.40 (4.17) |
|  | AMNART | 29.01 (7.81) |  | 35.39 (7.91) |
|  | Animal Naming Test | 14.89 (5.20) |  | 22.465 (5.73) |
| **El Haj et al. (2018)** | **Working memory** |  |  |  |
|  | Forward span | 5.13 (1.03) |  | 6.70 (1.54) |
|  | Backward span | 3.75 (1.15) |  | 4.74 (1.72) |
|  | **Verbal fluency** |  |  |  |
|  | Letter "P" | 16.75 (5.88) |  | 23.22 (4.50) |
|  | **Inhibition** |  |  |  |
|  | Stroop | 58.13 (9.59) |  | 35.78 (9.65) |
|  | **Depression** |  |  |  |
|  | HADS | 8.21 (1.28) |  | 6.74 (2.44) |
| **Huppert et al. (1993)** | **Intelligence** | **Minimal dementia M(range)** | **Mild/moderate dementia M(range)** | **Controls M(range)** |
|  | NART Number correct | 17.9 (6-32) | 20.9 (8-34) | 20.3 (6-36) |
|  | NART IQ | 101.3 (91-113) | 103.6 (93-114) | 103.1 (91-116) |
|  | **Cognitive impairment** |  |  |  |
|  | CAMCOG score | 78.4 (67-95) | 62.3 (27-87) | 84.8 (69-101) |
| **Kamminga et al. (2013)** | **General cognitive abilities** |  |  |  |
|  | ACE-R | 68.5 (15.7) |  | 93.8 (3.3) |
|  | **Speed of processing** |  |  |  |
|  | TMT - A | 93.6 (93.5) |  | 40.7 (13.5) |
|  | **Verbal episodic memory** |  |  |  |
|  | RAVLT delayed recall | 4.2 (3.3) |  | 11.3 (2.3) |
|  | **Visuospatial episodic recall** |  |  |  |
|  | RCFT 3 min recall | 5.8 (5.2) |  | 18.9 (5.8) |
|  | **Non-verbal recognition** |  |  |  |
|  | Doors A | 7.8 (2.6) |  | 10.4 (1.8) |
|  | **Attention/short-term working memory** |  |  |  |
|  | Digit Span Total | 12.1 (2.7) |  | 20.4 (4.7) |
|  | **Mental flexibility** |  |  |  |
|  | TMT B-A | 121.5 (69.2) |  | 43.6 (18.4) |
|  | **Verbal fluency** |  |  |  |
|  | Letter fluency (FAS) | 27.6 (19.6) |  | 44.5 (11.2) |
|  | **Inhibition** |  |  |  |
|  | Hayling Total | 3.7 (1.6) |  | 6.0 (0.8) |
|  | **Verbal semantic performance** |  |  |  |
|  | Naming (subscales of the SydBat) | 19.8 (4.9) |  | 24.9 (2.0) |
|  | Comprehension (subscales of the SydBat) | 25 (4.6) |  | 28 (2.5) |
| **Kinsella et al., 2007** | **Study 1** |  |  |  |
|  | **Intelligence Measure** |  |  |  |
|  | NART IQ | 104.07 (12.51) |  | 111.01 (6.68) |
|  | **Study 2** |  |  |  |
|  | **Intelligence Measure** |  |  |  |
|  | NART IQ | 103.13 (9.43) |  | 111.57 (7.82) |
|  | **Retrospective memory** |  |  |  |
|  | HVLT-R (DI) | 5.00 (2.78) |  | 9.56 (2.45) |
|  | **Shifting** |  |  |  |
|  | TMT B-A | 161.44 (61.76) |  | 91.31 (55.76) |
|  | **Working memory** |  |  |  |
|  | Digit span (tot) | 7.15 (1.54) |  | 9.28 (1.99) |
| **Lecouvey et al. (2019)** | **Retrospective episodic memory** |  |  |  |
|  | RL-Rl16 | 10.70 (7.50) |  | 26.30 (10.04) |
|  | **Semantic memory** |  |  |  |
|  | DO80 | 73.57 (5.14) |  | 79.40 (0.83) |
|  | **Fluency** |  |  |  |
|  | Categorial fluency test | 12.79 (6.15) |  | 28.93 (6.64) |
|  | Lexical fluency test | 11.07 (4.27) |  | 26.00 (7.94) |
|  | **Inhibition** |  |  |  |
|  | Stroop reading | 246.90 (141.90) |  | 76.87 (44.29) |
|  | Stroop interference | 2.00 (1.83) |  | 1.01 (0.73) |
|  | **Shifting** |  |  |  |
|  | TMT B | 117 (183.72) |  | 102 (38.65) |
|  | **Planning** |  |  |  |
|  | Zoo map test | 1.44 (1.09) |  | 2.33 (0.62) |
|  | **Time estimation** | 132.50 (160.40) |  | 42.48 (24.97) |
| **Lee et al. (2016)** | Associate Memory | 7.5 (1.13) |  | 13.0 (1.00) |
|  | Selective Reminding Test | 45.2 (0.9) |  | 47.9 (0.80) |
|  | Forward Digit Span | 6.5 (0.26) |  | 6.9 (0.23) |
|  | Backward Digit Span | 4.8 (0.31) |  | 4.9 (0.27) |
|  | TMT - A | 45.9 (3.9) |  | 36.3 (3.50) |
|  | TMT - B | 120.4 (10.0) |  | 92.6 (8.80) |
|  | WMS Logical Memory | 7.7 (1.07) |  | 13.7 (0.94) |
|  | Letter Number Sequencing | 6.7 (0.78) |  | 8.2 (0.69) |
| **Martins & Damasceno (2008)** | **Control tests** |  |  |  |
|  | Visual perception | 8.1 (1.3) |  | 9.0 (0.9) |
|  | **Retrospective episodic memory** |  |  |  |
|  | RAVLT recall | 1.1 (1.6) |  | 6.65 (2.6) |
|  | **Attention** |  |  |  |
|  | Digit span backwards | 3.3 (0.7) |  | 4.0 (0.9) |
|  | **Executive function** |  |  |  |
|  | TMT B | 12.3 (5.7) |  | 21.0 (6.0) |
| **Massa et al. (2020)** | **Executive functions** |  |  |  |
|  | TMT - B | 186.8 (112.1) |  | 129.9 (70.6) |
|  | Stroop Color | 33.7 (7.8) |  | 41.4 (9.0) |
|  | Stroop Color-Word | 12.5 (5.6) |  | 18.1 (5.9) |
|  | **Attention** |  |  |  |
|  | TMT - A | 59.4 (14.2) |  | 54.5 (25.0) |
|  | Digit Symbol | 26.2 (6.6) |  | 37.4 (10.3) |
|  | **Working Memory** |  |  |  |
|  | Corsi Span | 4.2 (0.6) |  | 4.6 (0.8) |
|  | Digit Span | 5.6 (0.7) |  | 5.5 (0.8) |
|  | **Verbal episodic memory** |  |  |  |
|  | RAVLT - immediate recall | 25.3 (8.7) |  | 40.0 (10.6) |
|  | RAVLT - delayed recall | 2.6 (2.0) |  | 8.0 (3.2) |
|  | Babcock Story Recall | 7.0 (4.9) |  | 15.0 (4.1) |
|  | **Visuospatial abilities** |  |  |  |
|  | Clock Drawing Test | 0.8 (1.8) |  | 0.2 (1.0) |
|  | Figure copy, simple | 10.0 (1.1) |  | 9.8 (1.2) |
|  | Figure copy with guiding landmarks | 67.1 (6.1) |  | 68.6 (2.0) |
|  | **Language** |  |  |  |
|  | Semantic fluency | 29.9 (7.6) |  | 44.0 (10.6) |
|  | Phonemic fluency | 32.4 (11.4) |  | 35.9 (9.6) |
|  | **Depression** |  |  |  |
|  | Geriatric Depression Scale | 2.5 (2.3) |  | 2.5 (1.7) |
| **Maylor et al. (2002)** | **Study 1** |  |  |  |
|  | Digit span | 5.35 (1.34) |  | 7.08 (1.37) |
|  | Sentence span | 2.92 (0.97) |  | 4.39 (0.56) |
|  | Free recall | 19.46 (5.86) |  | 25.93 (4.03) |
|  | **Study 2** |  |  |  |
|  | Digit span | 5.30 (0.79) |  | 6.75 (0.96) |
|  | Sentence span | 2.83 (1.16) |  | 4.23 (0.95) |
|  | Free recall | 19.17 (3.67) |  | 26.80 (5.37) |
| **Shelton et al. (2016)** | Associate memory | 6.5 (4.8) |  | 12.9 (4.8) |
|  | Selective reminding test | 45.4 (3.2) |  | 47.9 (0.3) |
|  | Forward digit span | 6.4 (1.1) |  | 6.8 (1.0) |
|  | Backward digit span | 4.7 (1.1) |  | 4.8 (1.4) |
|  | TMT A (sec) | 44.7 (17.1) |  | 36.4 (4.3) |
|  | TMT B (sec) | 123.9 (46.3) |  | 92.7 (40.9) |
|  | WMS Logical Memory | 7.2 (4.3) |  | 13.6 (3.2) |
|  | WMS Letter Number Sequencing | 6.7 (2.7) |  | 8.2 (3.5) |
| **Thompson et al. (2010)** | **Cognitive functions** |  |  |  |
|  | Visual Span | 8.10 (3.92) |  | 11.86 (3.58) |
|  | Tower of London (Excess moves) | 16.91 (18.04) |  | 7.39 (5.47) |
| **Troyer & Murphy (2007)** | Vocabulary SS | 10.8 (2.0) |  | 13.8 (2.9) |
|  | Digit span SS | 10.5 (2.9) |  | 11.5 (2.9) |
|  | HVLT immediate recall SS | 4.8 (1.9) |  | 10.4 (1.8) |
|  | BVMT immediate recall SS | 3.8 (1.8) |  | 9.7 (2.5) |
|  | Rey figure SS | 7.5 (2.3) |  | na |
|  | Boston naming SS | 6.5 (4.3) |  | na |
|  | TMT B SS | 5.3 (3.6) |  | 12.1 (2.5) |
| **Tse et al., 2015** | ADAS-Cog total error score | 12.31 (3.68) |  | 7.49 (2.27) |
|  | Verbal fluency item generation in 30 s | 24.11 (5.82) |  | 27.92 (5.85) |
|  | Verbal fluency item generation in 60 s | 33.56 (8.28) |  | 39.6 (8.36) |
|  | Verbal fluency intrusion errors | 0.16 (0.46) |  | 0.16 (0.49) |
|  | Forward/backward digit span | 6.82 (1.3) |  | 7.55 (1.13) |
|  | Forward/backward visual span | 3.67 (0.91) |  | 3.99 (0.88) |
| **Zhuang et al. (2021)** | FAB total score | 10.36 (2.79) |  | 13.65 (1.94) |
|  | Digit Span Forward | 9.55 (2.11) |  | 11.94 (2.17) |
|  | Digit Span Backward | 4.59 (1.56) |  | 6.81 (2.73) |
|  | Symbol Digit Modality Test - Correct | 19.09 (9.46) |  | 34.00 (14.53) |
|  | Symbol Digit Modality Test- Error | 1.27 (2.12) |  | 0.84 (1.10) |

^1^References to neuropsychological tasks are not reported because the authors referred to different versions of the tasks; please refer to the specific articles for the appropriate references.

*Note:* RAVTL = Rey Auditory Verbal Learning Task; RCFT = Rey Complex Figure; WMS = Wechsler Mental Scale; AMNART American version of the Adult Reading Test. TMT = Trial Making Test; SYDBAT = The Sydney Language Battery; HADS = Hospitalised Anxiety and Depression Scale; CAMCOG = Cambridge Cognitive Examination; NART = The National Adult Reading Test; Doors A = subtest from the Doors and People test; HVLT-R = Hopkins Verbal Learning Test–Revised Language Battery; FAB = Frontal Assessment Battery;

SS= age scaled score; na = Not Available.
