# Supplementary material for: Time-Based and Event-Based Prospective Memory in Mild Cognitive Impairment and Alzheimer’s Disease Patients: A Systematic Review and Meta-analysis
Source: Neuropsychol Rev. 2023 Nov 14;35(1):102–25. doi: 10.1007/s11065-023-09626-y (PMC11965160; doi:10.1007/s11065-023-09626-y)
Supplement: Supplementary file 2 — Supplementary file2 (DOCX 42 KB) [file 11065_2023_9626_MOESM2_ESM.docx]

**Supplementary 2:** Tasks included in the neuropsychological evaluation for MCI and controls.

|  | **Cognitive function^1^** (task used) | **MCI Patients M (SD)** |  | **Controls M (SD)** |
| --- | --- | --- | --- | --- |
| **Beaver et al. (2017)** | **Memory Assessment Scale:** |  |  |  |
|  | Activity content memory | 11.06 (2.10) |  | 12.38 (1.91) |
|  | MAS prose memory | 4.69 (1.31) |  | 6.01 (1.35) |
|  | Temporal order memory | 14.65 (6.43) |  | 10.35 (5.35) |
|  | **Functional Status Measures** |  |  |  |
|  | **Questionnaire** |  |  |  |
|  | IADL-C self | 49.08 (24.43) |  | 38.99 (12.72) |
|  | IADL-C KI | 47.40 (25.72) |  | 33.33 (8.18) |
|  | **Direct observation** |  |  |  |
|  | Eight-activities | 17.38 (5.87) |  | 14.27 (3.55) |
|  | Day out task | 15.00 (4.08) |  | 13.04 (2.99) |
|  | **Performance-based** |  |  |  |
|  | EPT | 18.82 (3.89) |  | 21.04 (2.39) |
|  | OTDL-R | 20.16 (3.89) |  | 22.59 (3.06) |
| **Belmar et al. (2020)** |  |  |  |  |
|  | **Attention** |  |  |  |
|  | Cancellation | 5.39 (1.80) |  | 5.81 (1.48) |
|  | **Verbal episodic memory** |  |  |  |
|  | LM-immediate | 0.46 (0.15) |  | 0.57 (0.15) |
|  | LM-delayed | 0.48 (0.21) |  | 0.58 (0.13) |
|  | **Attention, mental flexibility** |  |  |  |
|  | TMTA | 61 (3.49) |  | 53 (16) |
|  | TMTB | 166 (82) |  | 131 (45) |
|  | **Questionnaire** |  |  |  |
|  | PRMQ-prospective | 11.32 (2.04) |  | 21.80 (5.13) |
|  | PRMQ-retrospective | 11.43 (2.93) |  | 20.22 (5.12) |
|  | **Subjective Memory Complaints** |  |  |  |
|  | SMC | 0.52 (0.16) |  | 0.11 (0.07) |
|  | **Depression Scale** |  |  |  |
|  | GDS | 0.32 (0.17) |  | 0.15 (0.11) |
| **Costa et al. (2011)** | **Depression Scale** |  |  |  |
|  | CDRS | 0.4 (0.2) |  | na |
|  | **Questionnaire** |  |  |  |
|  | ADL | 5.8 (0.5) |  | na |
|  | IADL | 7.0 (1.4) |  | na |
| **Costa et al. (2011)** | **Questionnaire** |  |  |  |
|  | ADL | 6.0 (0.0) |  | na |
|  | IADL | 7.6 (1.1) |  | na |
|  | **Episodic Memory** |  |  |  |
|  | Immediate recall Rey's word list | 34.00 (7.50) |  | na |
|  | Delayed recall Rey's words list | 6.20 (2.60) |  | na |
|  | Immediate prose recall | 3.80 (2.50) |  | na |
|  | Delayed prose recall | 3.50 (2.74) |  | na |
|  | **Short-term memory** |  |  |  |
|  | Digit span | 5.03 (0.98) |  | na |
|  | Corsi span | 4.47 (0.68) |  | na |
|  | **Executive functions** |  |  |  |
|  | Phonological verbal fluency | 30.58 (10.59) |  | na |
|  | MCST (Categories) | 4.68 (1.53) |  | na |
|  | MCST (perseverative errors) | 2.12 (2.88) |  | na |
|  | MCST (non-perseverative errors) | 7.61 (6.65) |  | na |
|  | **Language** |  |  |  |
|  | Token test | 33.67 (2.55) |  | na |
|  | Naming Aachener aphasia | 8.66 (0.48) |  | na |
|  | **Abstract reasoning** |  |  |  |
|  | Raven's matrices | 28.19 (4.35) |  | na |
|  | **Constructive praxis** |  |  |  |
|  | Copy of drawings | 9.96 (1.36) |  | na |
|  | Copy of drawings with landmarks | 67.63 (2.61) |  | na |
|  | **Attention** |  |  |  |
|  | Attentive matrices | 36.95 (7.34) |  | na |
| **Costa et al., 2015** |  | **Single domain aMCI** | **Multiple domain aMCI** | **Controls** |
|  | **Global cognitive functioning** |  |  |  |
|  | Progressive matrices | 30.6 (4.4) | 27.9 (5.1) | 29.3 (4.6) |
|  | **Executive functions** |  |  |  |
|  | MCST-categories | 5.4 (1.2) | 4.5 (1.2) | 5.7 (0.6) |
|  | MCST-perseverative errors | 3.18 (4.5) | 4.3 (3.3) | 1.3 (2.4) |
|  | **Phonemic verbal fluency** | 31.7 (8.2) | 33.0 (10.0) | 31.3 (7.8) |
|  | **Episodic memory** |  |  |  |
|  | Word List Learning-immediate recall | 30.7 (6.5) | 33.8 (9.3) | 41.1 (7.2) |
|  | Word List Learning-delayed recall | 5.0 (2.2) | 6.1 (2.7) | 7.3 (2.2) |
|  | Prose Memory-immediate recall | 4.5 (2.1) | 5.4 (1.3) | 5.9 (1.1) |
|  | Prose Memory-delayed recall | 4.1 (2.3) | 5.5 (1.3) | 5.9 (1.1) |
|  | Rey’s Fig.-immediate reproduction | 13.5 (9.9) | 12.2 (6.9) | 16.0 (6.9) |
|  | Rey’s Fig.-delayed reproduction | 13.5 (9.6) | 10.6 (6.2) | 16.2 (5.8) |
|  | **Short-term memory** |  |  |  |
|  | Digit Span forward | 5.8 (0.9) | 5.3 (1.1) | 5.5 (1.0) |
|  | Corsi Block test forward | 4.5 (0.4) | 3.9 (0.6) | 5.0 (0.7) |
|  | **Visual-constructional praxis** |  |  |  |
|  | Rey’s Fig. copy | 30.2 (8.7) | 30.5 (7.6) | 31.5 (3.7) |
|  | Copy of drawings | 9.9 (1.3) | 9.7 (1.7) | 9.9 (1.0) |
|  | Copy of drawings with landmarks | 67.6 (2.8) | 66.3 (3.8) | 68.4 (1.5) |
| **Delprado et al. (2012)** | **Global cognitive functioning** |  |  |  |
|  | WTAR predicted IQ | 109.40 (6.74) |  | 108.80 (8.17) |
|  | **Retrospective memory** |  |  |  |
|  | CVLT-II (long-delay) | 3.63 (3.34) |  | 10.56 (2.91) |
|  | **Working memory** |  |  |  |
|  | Digit span backwards (WAIS-III) | 6.27 (1.75) |  | 6.80 (2.24) |
|  | DKEFS Letter fluency | 37.55 (11.39) |  | 39.07 (12.18) |
|  | DKEFS Category fluency | 31.73 (7.03) |  | 38.04 (8.00) |
|  | DKEFS Switching fluency | 10.24 (2.76) |  | 12.82 (3.06) |
|  | **Switch** |  |  |  |
|  | TMT B-A | 88.50 (76.25) |  | 55.00 (39.33) |
|  | **Divide attention** |  |  |  |
|  | TEA dual task | 1.91 (3.02) |  | 1.18 (2.18) |
| **Karantzoulis et al. (2009)** | **Dementia Rating Scale (SS)** |  |  |  |
|  | Attention | 11.93 (0.29) |  | 12.41 (0.24) |
|  | Inattention/perseveration | 10.22 (0.37) |  | 11.26 (0.27) |
|  | Construction | 9.89 (0.11) |  | 10.00 (0.00) |
|  | Conceptualization | 11.48 (0.29) |  | 11.89 (0.25) |
|  | Memory | 8.59 (0.70) |  | 12.04 (0.31) |
|  | Total | 10.81 (0.41) |  | 13.22 (0.37) |
|  | Total recall (trials 1-3) | 8.22 (1.07) |  | 12.81 (0.57) |
|  | Delay | 7.56 (1.72) |  | 11.67 (0.37) |
|  | % Retention | 6.48 (0.82) |  | 11.22 (0.52) |
|  | Recognition (hits-false alarms) | 9.04 (0.65) |  | 11.55 (0.28) |
|  | Immediate | 7.67 (0.66) |  | 11.78 (0.42) |
|  | Delay | 7.22 (0.63) |  | 12.00 (0.40) |
|  | % Retention | 7.89 (0.53) |  | 11.56 (0.43) |
|  | Immediate | 8.93 (0.44) |  | 12.44 (0.60) |
|  | Delay | 10.0 (0.49) |  | 12.93 (0.29) |
|  | Copy | 9.26 (0.41) |  | 10.33 (0.45) |
|  | Immediate | 7.11 (0.62) |  | 11.63 (0.49) |
|  | Delay | 6.92 (0.55) |  | 10.88 (0.54) |
|  | **Wechsler Adult Intelligence Scale-Revised** |  |  |  |
|  | Block design | 10.93 (0.50) |  | 12.67 (0.60) |
|  | **Boston Naming Test (SS)** | 10.26 (0.42) |  | 12.07 (0.59) |
|  | **Shipley Vocabulary Test (SS)** | 13.63 (0.31) |  | 14.22 (0.35) |
|  | **Questionnaire** | 36.96 (2.24) |  | 41.00 (2.15) |
|  | IADL (raw scores) | 0.70 (0.31) |  | 0.00 (0.00) |
| **Kinsella et al. (2016)** | **Global cognitive functioning** |  |  |  |
|  | WTAR: predicted IQ | 108.83 (7.37) |  | 109.56 (7.84) |
|  | **Memory measures** |  |  |  |
|  | HVLT-R delayed recall | 0.94 (1.63) |  | 9.09 (2.12) |
|  | Logical memory delayed recall | 7.67 (6.73) |  | 23.27 (7.56) |
|  | Verbal paired delayed recall | 3.85 (2.27) |  | 7.50 (1.88) |
|  | Rey figure delayed recall | 7.58 (5.18) |  | 16.37 (5.68) |
| **Lajeunesse et al. (2020)** |  |  |  |  |
|  | **Executive composite score** | -0.44 (0.73) |  | 0.44 (0.73) |
|  | **Depression Scale** |  |  |  |
|  | GDS | 7.36 (5.36) |  | 3.92 (3.76) |
| **Massa et al. (2020)** | **Executive functions** |  |  |  |
|  | TMT B | 186.8 (112.1) |  | 129.9 (70.6) |
|  | Stroop Color | 33.7 (7.8) |  | 41.4 (9.0) |
|  | Stroop Color-Word | 12.5 (5.6) |  | 18.1 (5.9) |
|  | **Attention** |  |  |  |
|  | TMT A | 59.4 (14.2) |  | 54.5 (25.0) |
|  | Digit Symbol | 26.2 (6.6) |  | 37.4 (10.3) |
|  | **Working memory** |  |  |  |
|  | Corsi Span | 4.2 (0.6) |  | 4.6 (0.8) |
|  | Digit Span | 5.6 (0.7) |  | 5.5 (0.8) |
|  | **Verbal episodic memory** |  |  |  |
|  | RAVLT immediate recall | 25.3 (8.7) |  | 40.0 (10.6) |
|  | RAVLT delayed recall | 2.6 (2.0) |  | 8.0 (3.2) |
|  | Babcock Story Recall | 7.0 (4.9) |  | 15.0 (4.1) |
|  | **Visuospatial abilities** |  |  |  |
|  | Clock Drawing Test | 0.8 (1.8) |  | 0.2 (1.0) |
|  | Figure copy, simple | 10.0 (1.1) |  | 9.8 (1.2) |
|  | Figure copy with guiding landmarks | 67.1 (6.1) |  | 68.6 (2.0) |
|  | **Language** |  |  |  |
|  | Semantic fluency | 29.9 (7.6) |  | 44.0 (10.6) |
|  | Phonemic fluency | 32.4 (11.4) |  | 35.9 (9.6) |
|  | **Depression Scale** |  |  |  |
|  | GDS | 2.5 (2.3) |  | 2.5 (1.7) |
| **Niedzwienska et al. (2017)** | **Episodic Memory** |  |  |  |
|  | WMS Logical memory (immediate) | 21.71 (8.92) |  | 42.80 (11.05) |
|  | WMS Logical Memory (delayed) | 7.85 (8.03) |  | 25.52 (7.51) |
|  | WMS Verbal Paired Associates (immediate) | 6.50 (6.31) |  | 16.85 (7.14) |
|  | WMS Verbal Paired Associates (delayed) | 1.94 (1.97) |  | 5.44 (2.42) |
|  | HVLT (immediate 1) | 3.65 (1.59) |  | 6.58 (1.63) |
|  | HVLT (immediate 2) | 5.21 (1.61) |  | 8.53 (1.82) |
|  | HVLT (immediate 3) | 6.27 (1.46) |  | 9.51 (1.77) |
|  | HVLT (delayrd) | 2.77 (3.11) |  | 8.49 (2.60) |
|  | **Short-term memory** |  |  |  |
|  | WMS Digit Span (Forward) | 10.18 (2.24) |  | 10.33 (2.55) |
|  | WMS Digit Span (Backward) | 6.71 (2.25) |  | 7.44 (2.38) |
|  | **Attention and executive functions** |  |  |  |
|  | Verbal fluency: Letters | 33.65 (13.37) |  | 41.07 (13.42) |
|  | Verbal fluency: Category | 11.85 (4.43) |  | 17.76 (5.56) |
|  | TMTA | 45.07 (11.55) |  | 36.48 (11.23) |
|  | TMTB | 124.54 (57.06) |  | 81.02 (33.54) |
| **Pereira et al. (2015)** | **Memory** |  |  |  |
|  | Logical Memory | 8.57 (5.27) |  | 14.79 (3.51) |
|  | **Visual attention and task switching** |  |  |  |
|  | TMT (B-A) | 144.32 (90.52) |  | 67.91 (46.56) |
|  | **Questionnaires** |  |  |  |
|  | SWLS | 26.59 (6.29) |  | 23.3 (6.53) |
|  | SMC | 9.13 (3.91) |  | 8.11 (4.00) |
|  | GDS | 9.54 (4.68) |  | 7.97 (5.23) |
| **Schmitter-Edgecombe et al. (2009)** |  | **NonAmnestic MCI** | **Amnestic MCI** | **Controls** |
|  | **Attention/speed** |  |  |  |
|  | SDMT written | 38.87 (14.40) | 36.63 (9.46) | 44.50 (10.11) |
|  | SDMT oral | 44.00 (14.82) | 42.38 (10.79) | 50.86 (11.49) |
|  | Trails A (time) | 41.07 (12.77) | 43.81 (11.60) | 38.93 (12.50) |
|  | **Verbal memory** |  |  |  |
|  | RAVLT trials 1–5 | 46.73 (9.54) | 34.15 (8.16) | 48.29 (10.40) |
|  | RAVLT imm delay | 9.60 (3.54) | 4.67 (3.03) | 9.02 (3.05) |
|  | RAVLT long delay | 9.67 (2.77) | 4.51 (3.15) | 8.83 (3.21) |
|  | **Language** |  |  |  |
|  | BNT | 55.73 (3.55) | 52.77 (9.00) | 56.55 (2.94) |
|  | D-KEFS category fluency | 38.47 (8.86) | 32.37 (7.57) | 41.31 (8.97) |
|  | **Executive** |  |  |  |
|  | D-KEFS letter fluency | 39.47 (12.74) | 36.81 (13.68) | 43.10 (11.73) |
|  | Trials B (time) | 117.93 (45.62) | 138.80 (58.96) | 89.95 (34.51) |
|  | WAIS-III Letter–Number Sequencing subtest | 8.00 (2.75) | 8.33 (2.06) | 9.60 (2.43) |
| **Tam & Schmitter-Edgecombe (2013)** | **Verbal intelligence** |  |  |  |
|  | SILS Vocabulary | 33.96 (6.91) |  | 36.00 (5.59) |
|  | **Memory** |  |  |  |
|  | RAVLT List Learning | 34.75 (6.52) |  | 47.54 (9.79) |
|  | RAVLT Short Delay | 4.92 (2.99) |  | 9.38 (2.83) |
|  | RAVLT Long Delay | 5.04 (2.84) |  | 9.21 (3.19) |
|  | 7/24 Learning | 23.38 (6.22) |  | 30.17 (4.77) |
|  | 7/24 Short Delay | 4.30 (1.96) |  | 5.67 (2.06) |
|  | 7/24 LD | 4.33 (1.99) |  | 5.58 (1.79) |
|  | **Attention/speed** |  |  |  |
|  | SDMT written | 33.67 (8.43) |  | 42.38 (11.54) |
|  | Trails A (time) | 45.35 (9.37) |  | 39.71 (13.18) |
|  | **Working memory** |  |  |  |
|  | WAIS-III Letter–Number Sequencing subtest | 7.79 (2.17) |  | 10.01 (2.65) |
|  | **Executive Functions** |  |  |  |
|  | D-KEFS Letter Fluency subtest | 36.96 (14.25) |  | 37.25 (10.81) |
|  | Trails B-A/A | 2.15 (0.97) |  | 1.41 (0.85) |
|  | D-KEFS Design Fluency subtest | 20.83 (6.86) |  | 24.21 (6.93) |
|  | CLOX 1 | 11.79 (2.19) |  | 12.92 (2.26) |
|  | **Language** |  |  |  |
|  | BNT | 51.00 (9.10) |  | 56.46 (2.96) |
|  | D-KEFS Category Fluency subtest | 35.46 (8.52) |  | 40.58 (8.94) |
| **Thompson et al. (2010)** | **Cognitive functions** |  |  |  |
|  | Visual Span | 9.96 (3.57) |  | 8.10 (3.92) |
|  | Tower of London (Excess moves) | 8.33 (6.11) |  | 16.91 (18.04) |
| **Troyer & Murphy (2007)** | Vocabulary SS | 13.5 (2.8) |  | 13.8 (2.9) |
|  | Digit span SS | 12.2 (3.0) |  | 11.5 (2.9) |
|  | HVLT immediate recall SS | 6.8 (2.1) |  | 10.4 (1.8) |
|  | BVMT immediate recall SS | 5.0 (2.5) |  | 9.7 (2.5) |
|  | Rey-Osterreith figure copy SS | 8.2 (2.8) |  | na |
|  | Boston naming SS | 10.0 (3.2) |  | na |
|  | TMT B SS | 10.1 (2.5) |  | 12.1 (2.5) |
| **Wang et al. (2012)** |  | **NonAmnestic MCI** | **Amnestic MCI** | **Controls** |
|  | **Non-memory test** |  |  |  |
|  | CFT-copy | 29.83 (6.05) | 30.57 (5.67) | 33.37 (2.33) |
|  | SDMT-correct | 32.82 (11.40) | 37.84 (11.15) | 45.22 (11.03) |
|  | TMT-A | 69.14 (27.88) | 70.33 (26.73) | 48.08 (13.53) |
|  | TMT-B | 206.95 (73.64) | 201.70 (79.56) | 136.48 (48.10) |
|  | Stroop Color-Word Test-C-time (s) | 97.02 (29.02) | 98.27 (33.07) | 74.35 (20.63) |
|  | Stroop Color-Word Test-C-correct | 40.84 (6.94) | 40.63 (8.21) | 46.89 (2.94) |
|  | Similarity test | 11.85 (6.84) | 10.61 (5.42) | 16.19 (3.53) |
|  | CESDT | 10.87 (9.34) | 12.16 (9.91) | 10.35 (8.97) |
|  | **Other memory tests** |  |  |  |
|  | Animal Fluency test | 14.31 (4.37) | 13.66 (3.93) | 15.59 (3.73) |
|  | Boston naming test | 21.16 (4.44) | 20.68 (4.10) | 23.96 (3.69) |
|  | SDMT-accidental memory | 2.88 (2.40) | 1.64 (2.19) | 3.95 (2.59) |
|  | ST-working memory | 2.95 (1.92) | 2.10 (2.03) | 4.00 (2.01) |
|  | Judgment of Confidence | 3.15 (2.85) | 4.34 (3.29) | 2.95 (2.93) |
| **Zhou et al. (2012)** | **Questionnaire** |  |  |  |
|  | ADL | 15.31 (1.92) |  | 14.43 (0.98) |
|  | **Memory** |  |  |  |
|  | Directed Memory | 10.77 (3.15) |  | 16.65 (2.41) |
|  | RCPTC | 2.00 (2.72) |  | 9.77 (3.82) |
|  | Story recall | 4.65 (1.46) |  | 8.40 (2.02) |
|  | **Executive function** |  |  |  |
|  | TMT-B | 89.07 (41.20) |  | 86.10 (39.81) |
|  | Digit-span backward | 4.38 (1.10) |  | 4.52 (1.83) |
|  | Stroop Color-Word Test | 48.24 (18.33) |  | 34.61 (9.66) |

^1^References to neuropsychological tasks are not reported because the authors referred to different versions of the tasks; please refer to the specific articles for the appropriate references.

*Note:* IADL-C = Instrumental Activities of Daily Living: Compensation Scale (KI = knowledgeable informant); EPT = Everyday Problems Test; OTDL-R = Revised Observed Test of Daily Living; LM = Logical memory – story a (Wechsler Memory Scale-III); CESDT, Center for Epidemiologic Studies Depression test; CFT, Rey-Osterrieth Complex Figure Test; WTAR = Wechsler Test of Adult Reading; SDMT = Symbol Digit Modalities Test; TMT = Trail Making Test; WMS = Wechsler Memory Test; HVLT = Hopkins Verbal Learning Test; SWLS = Satisfaction with Life Scale; SMC = Subjective Memory Complaints Scale; CDRS = Clinical Dementia Rating Scale; AVLT = Auditory Verbal Learning Test; AVLT-I = sum of three instant memory scores; RAVLT = Rey Auditory Learning Test; BNT = Boston Naming Test; SILS = Shipley Institute of Living Scale; WAIS–III = Wechsler Adult Intelligence Scale–Third Edition; D-KEFS = Delis–Kaplan Executive Functioning Scale; PRMQ = Prospective and Retrospective Memory Questionnaire; GDS = Geriatric Depression Scale; MCST = Modified Card Sorting Test; CVLT-II = Californian Verbal Learning Test – Second Edition; RCPTC = Recall of the Connection between Portraits and Their Characteristics; SS= age scaled score.
